# Conformational Control of Fast Asparagine Deamidation in a Norovirus Capsid Protein

**DOI:** 10.1021/acs.biochem.2c00656

**Published:** 2023-02-21

**Authors:** Robert Creutznacher, Eric Schulze-Niemand, Patrick König, Vesna Stanojlovic, Alvaro Mallagaray, Thomas Peters, Matthias Stein, Mario Schubert

**Affiliations:** † Institute of Chemistry and Metabolomics, University of Lübeck, Ratzeburger Allee 160, 23562 Lübeck, Germany; ‡ Molecular Simulations and Design Group, Max Planck Institute for Dynamics of Complex Technical Systems, Sandtorstrasse 1, 39106 Magdeburg, Germany; § Department of Biosciences and Medical Biology, University of Salzburg, Hellbrunnerstrasse 34, 5020 Salzburg, Austria

## Abstract

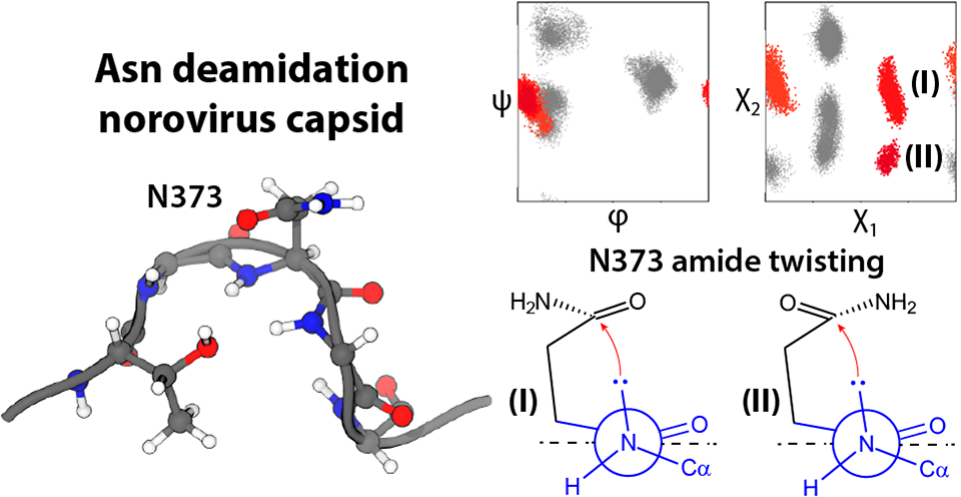

Accelerated spontaneous deamidation of asparagine 373 and subsequent conversion into an isoaspartate has been shown to attenuate the binding of histo blood group antigens (HBGAs) to the protruding domain (P-domain) of the capsid protein of a prevalent norovirus strain (GII.4). Here, we link an unusual backbone conformation of asparagine 373 to its fast site-specific deamidation. NMR spectroscopy and ion exchange chromatography have been used to monitor the deamidation reaction of P-domains of two closely related GII.4 norovirus strains, specific point mutants, and control peptides. MD simulations over several microseconds have been instrumental to rationalize the experimental findings. While conventional descriptors such as available surface area, root-mean-square fluctuations, or nucleophilic attack distance fail as explanations, the population of a rare *syn*-backbone conformation distinguishes asparagine 373 from all other asparagine residues. We suggest that stabilization of this unusual conformation enhances the nucleophilicity of the backbone nitrogen of aspartate 374, in turn accelerating the deamidation of asparagine 373. This finding should be relevant to the development of reliable prediction algorithms for sites of rapid asparagine deamidation in proteins.

## Introduction

**Scheme 1:**
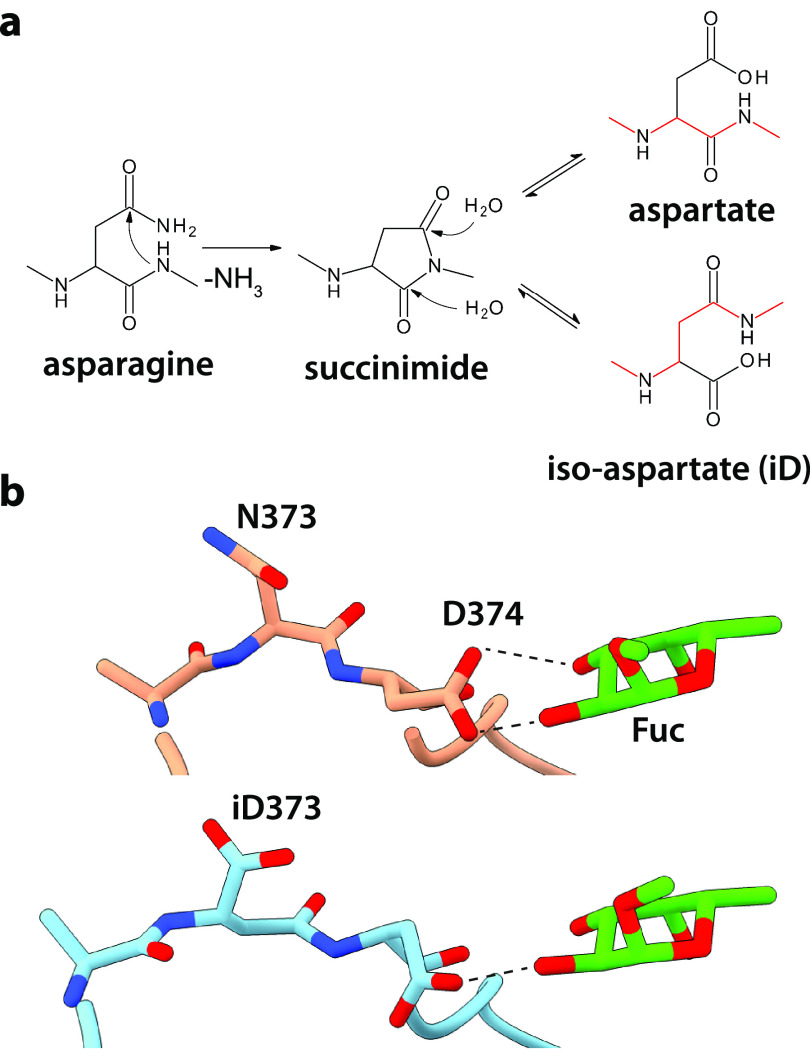
(a) Deamidation of Asn Residues, Which Can Produce Either Asp or isoAsp Residues; and (b) Structure of the Loop in GII.4 Saga P-Domains Harboring N373 before (Orange) and after (Blue) Deamidation The production of isoAsp residues results in the formation of an isopeptide bond in the protein backbone (red). PDB IDs 4X06, 6H9V, respectively. Reorientation of D374 after deamidation leads to a significant loss in binding affinity for l-fucose and l-fucose-containing glycans such as HBGAs.

Asparagine (Asn) residues in proteins and peptides often undergo spontaneous post-translational deamidation.^1−3^ Asn is converted either into aspartate (Asp) or into isoaspartate (isoAsp) via a succinimide intermediate (Scheme 1a). This reaction requires a nucleophilic attack of the backbone amide nitrogen of residue *i* + 1 onto the Asn side chain carbonyl carbon. As a result, an additional negative charge is created, and in the case of isoAsp, an isopeptide bond is formed in the protein backbone. The deamidation of Asn is irreversible, whereas Asp and isoAsp can interconvert in an equilibrium reaction and are typically present in a 3:1 ratio in model peptides.^4^ Deamidation reactions have mostly been described in the context of protein aging or degradation. Pharmaceutically relevant examples of a loss of function are therapeutic monoclonal antibodies that may lose antigen-binding capabilities upon spontaneous deamidation.^4−6^ Gain of function from Asn deamidation is rare but has been observed for a few proteins, e.g., the activation of a fibronectin-integrin binding site,^7^ or the stabilization of the bacterial enzyme MurA.^8^ We have previously observed deamidation of a surface-exposed Asn residue in the major capsid protein VP1 of a human norovirus (HuNoV). Asn373, located in the capsid’s protruding domain (P-domain), is not part of sequence motifs that have been reported to be prone to deamidation.^9−11^ With a half-life of 1.6 days at 37 °C this conversion is among the fastest reported so far and leads to the exclusive formation of an isoAsp residue.^12^

**Figure 1:**
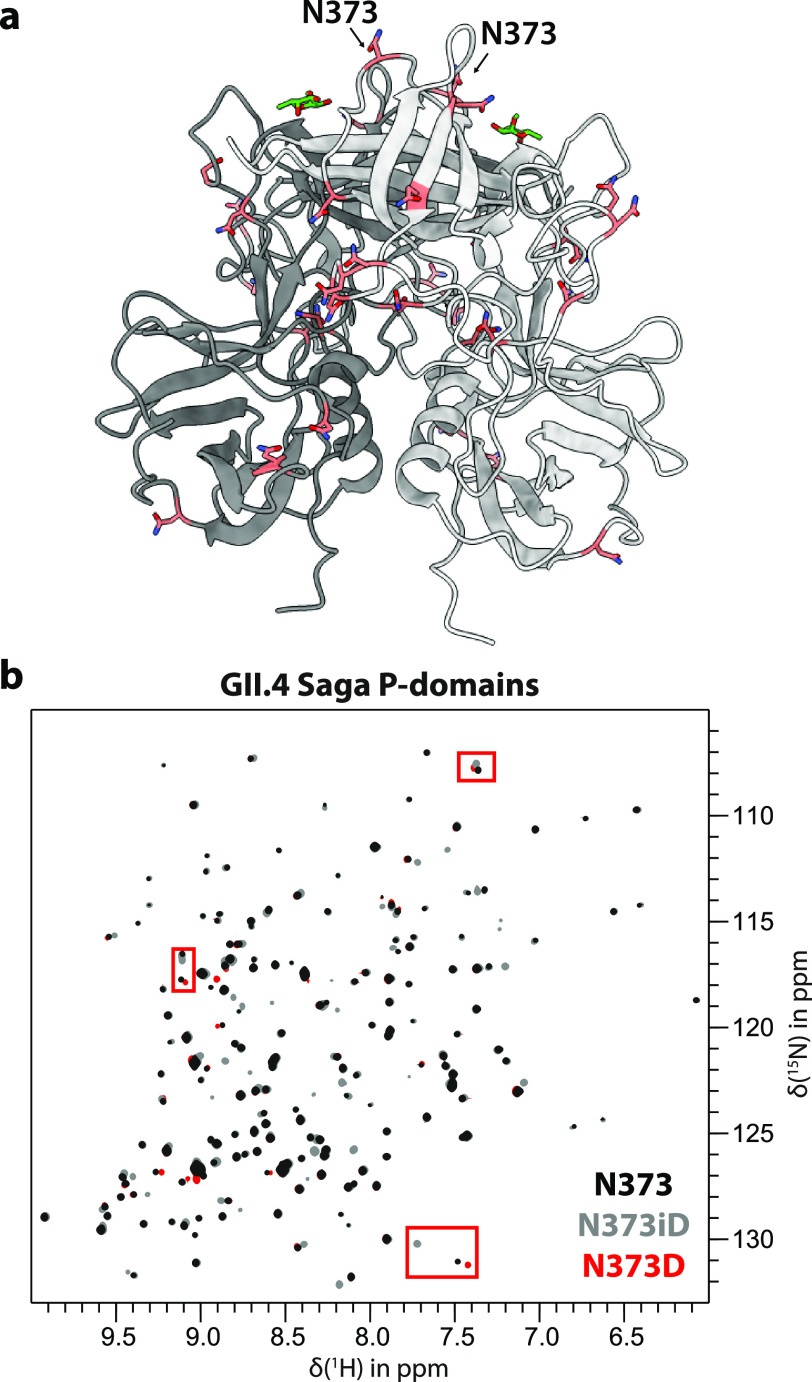
Selective deamidation of N373 in GII.4 NoV Saga P-domains. (a) Each monomer of the P-domain dimer contains 17 Asn residues, but only N373 undergoes deamidation (PDB 4X06). (b) ^15^N TROSY HSQC spectra of [*U*-^2^H,^15^N]-labeled GII.4 Saga P-domains reveal that the product of the deamidation reaction is exclusively isoAsp. Recombinantly introducing the N373D point mutant (red) produces characteristic signals that do not overlap with any of the wild type Asn (black) or isoAsp (gray) species’ signals (red boxes). These signals are never found in any of the spectra of the deamidating protein, indicating that the reaction equilibrium is strongly shifted toward isoAsp with Asp being below the limit of detection.

HuNoVs are nonenveloped RNA viruses responsible for an estimated 685 million cases of acute gastroenteritis every year (cf. https://www.cdc.gov/norovirus/trends-outbreaks/burden-US.html). Infection requires attachment of the virus to histo blood group antigens (HBGAs) on the surface of host cells via dimeric P-domains (P-dimers).^13−16^ The l-fucose residue present in all HBGAs serves as a minimal binding motif for the prevalent genogroup II, genotype 4 (GII.4) HuNoVs.^17^ The HBGA-binding pocket of GII.4 HuNoVs includes a critical aspartate residue, D374, that forms a bidentate hydrogen bond with two hydroxy groups of the l-fucose residue.^18,19^ In the GII.4 Saga strain, deamidation of the neighboring N373 and subsequent formation of an isoAsp residue impedes glycan recognition because it induces changes to the backbone conformation of this loop and affects the overall protein dynamics. This leads to the loss of a hydrogen bond essential for binding to l-fucose (Scheme 1b).^12,20^ N373 is highly conserved among GII.4 NoV strains, and we have confirmed that deamidation is not unique for GII.4 Saga but is also observed for P-domains of other GII.4 strains.^21^ Of note, fast deamidation is observed exclusively for N373 although Asn residues are abundant in the P-domain (Figure 1a).

Canonical sequence-based rules to predict sites of deamidation did not identify N373 to stand out. Peptide-based experiments have established that especially Asn residues followed by Gly and Ser show fast deamidation with half-life times at 37 °C between 1 and 13 days.^1,2^ Thus, deamidation of Asn in other sequence contexts has largely been disregarded. It is well established that deamidation rates of Asn residues in folded proteins are often much slower compared to rates found for corresponding short, unstructured peptides, suggesting that the three-dimensional fold protects the Asn residues from deamidation.^10,22,23^ In isolated cases the protein fold has been held responsible for substantially accelerating Asn deamidation.^8^ In these previous studies several parameters based on 3D structure models have been proposed to affect deamidation rates. It has been recognized that the Asn side chain must adopt a reactive conformation with the distance between the nucleophilic backbone nitrogen of residue *i* + 1 and the electrophilic carbonyl carbon being short enough to allow for a nucleophilic attack.^23^ It follows that the reaction rate should also depend on conformational flexibility of the polypeptide chain embedding the deamidation site. Consequently, high solvent accessibility, side chain flexibility, and accessibility of reactive conformations were highlighted as essential parameters.^23−25^ Free energy calculations using QM/MM methods suggested that certain backbone conformations of the *i* + 1 residue are linked with increased amide acidity which in turn favors nucleophilic attack and deamidation.^22^ That study concluded that proteins on average deamidate much slower than corresponding peptides due to conformational constraints imposed by secondary structure or hydrogen bonds that prevent access to reactive conformations.

It appears that the accurate prediction of accelerated deamidation in proteins is still a challenge. Confronted with the large number of potential structural descriptors, machine learning approaches condensing these parameters into a single model have been developed.^24,26,27^ Recently, prediction of Asn deamidation rates based on amino acid sequence and computed homology models has been reported to perform reasonably well for “conventional” deamidation sites (e.g., NG or NS) in IgGs.^27^ However, the prediction accuracy dropped for non-IgG proteins, and predicted Asn half-life times deviated substantially from experimental data especially for very fast and arguably the most important deamidation reactions. These puzzling but obvious discrepancies between experimental observations and predictions motivated our search for principles underlying the fast deamidation of human NoV P-domains^12^ as an interesting case study. We have shown that N373 (Figure 1a) is the only Asn residue undergoing fast deamidation in the P-domain, and moreover, only formation of isoaspartate (iD373) was observed. Subsequent HDX MS studies suggested that protein dynamics are linked to Asn deamidation.^20^ These observations led us to hypothesize that the combination of carefully designed NMR experiments in combination with molecular dynamics (MD) simulations may reveal a causal relationship between protein dynamics and fast deamidation of N373, eventually furnishing novel descriptors that would improve identification of labile Asn residues.

## Experimental Section

### Protein Biosynthesis and Purification

GII.4 Saga P-domains (amino acids 225–530, GenBank AB447457) and GII.4 VA387 P-domains (amino acids 225–529, GenBank AY038600) were synthesized and purified as described previously.^12,21^ The amino acids GPGS (Saga) or GP (VA387) were added to the N-terminus of the P-domain to provide a proteolytic cleavage site that separates the P-domains from the remainder of the His-tagged MBP fusion proteins. [*U*-^2^H,^15^N]-labeling was achieved by providing 3 g L^–1^ deuterated glucose (Deutero) and ^15^N ammonium chloride (Deutero) as sole carbon and nitrogen sources, respectively, during expression in D_2_O-based minimal media. Both proteins were subjected to an unfolding–refolding procedure to complete HD exchange for NMR studies.^12^ N373D, H297R, and N372E mutant proteins were generated using standard site-direct mutagenesis protocols as described elsewhere.^28,29^

### Ion Exchange Chromatography

**Figure 2:**
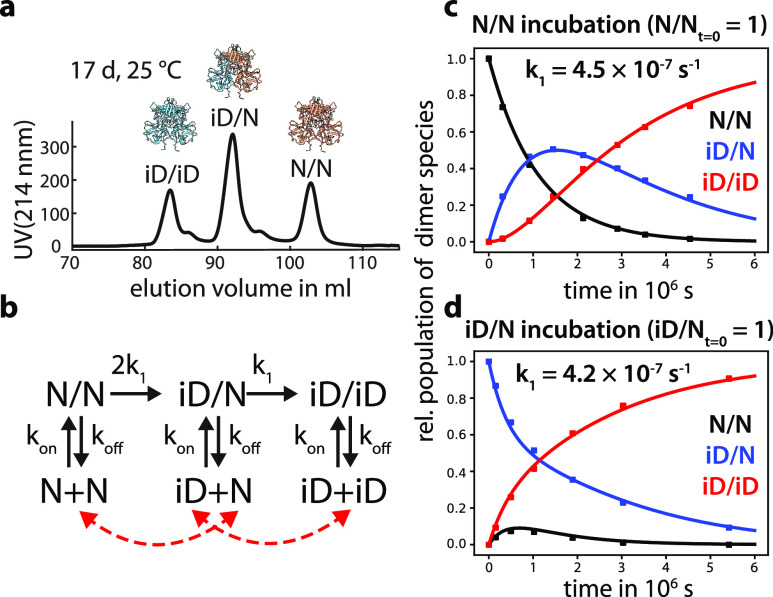
Deamidation reaction rate constant *k*_1_ of GII.4 Saga P-domains. (a) Proteins with different deamidation status can be separated via ion exchange chromatography after incubation at 25 °C. In the homodimeric P-domains, three major species can occur: N/N, iD/N, and iD/iD. (b) Kinetic model describing the deamidation process in the context of a homodimeric protein. Deamidation with the rate constant *k*_1_ is an irreversible process. Statistically, the first deamidation event is twice as likely as the second. Simultaneously, all species can dissociate into monomers with the dissociation rate constant *k*_off_ and reassemble into different dimer species (red arrows). (c) Purified N/N GII.4 Saga P-domains were incubated at 25 °C (pH 7.3), and the different dimer species were quantified by IEX. Using the experimental curves, numerical solution of the corresponding system of differential equations (eqs 4–8) yielded the deamidation reaction rate *k*_1_. (d) Repeating the experiment with isolated iD/N dimers highlights the importance of including dimer dissociation into the model as substantial amounts of N/N dimers can reassemble.

**Figure 3:**
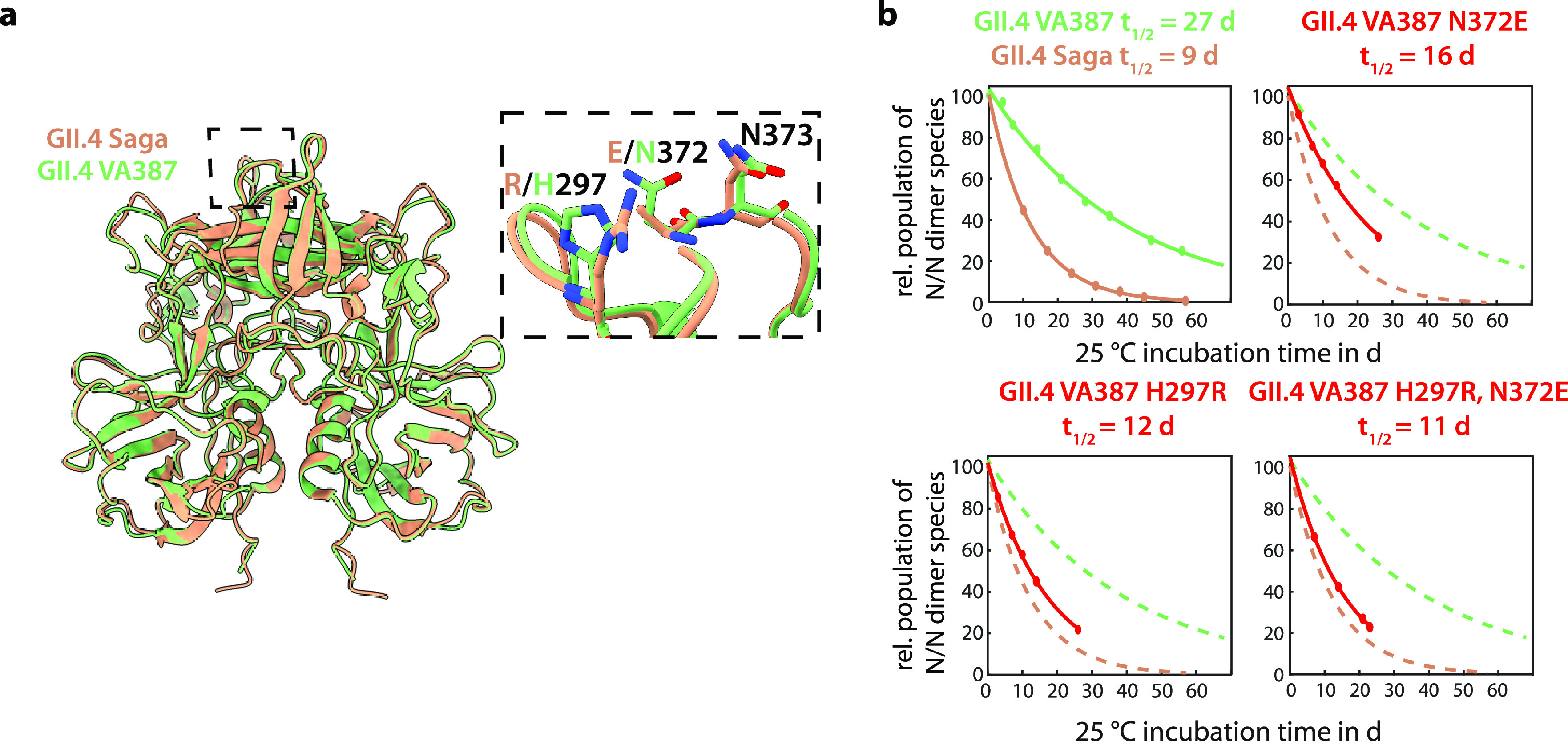
Deamidation of GII.4 VA387 P-domains. (a) Structural alignment of GII.4 VA387 P-domain dimers (green, PDB code 2OBT) with GII.4 Saga P-domains (orange, PDB code 4X06). The deamidation site and nearby amino acid substitutions are highlighted. (b) P-domain incubation at 25 °C and IEX chromatography yield N373 half-life times *t*_1/2_. Deamidation of Saga P-domains is three times faster than that of VA387 P-domains. Mutating VA387 amino acids close to N373 into their Saga counterparts reveals a strong influence of R297 on the deamidation rate of N373. Representative IEX chromatograms are shown in Figure S7.

P-domain species with different deamidation status were separated with a 6 mL Resource S cation exchange column (Cytiva) as described previously.^12^ For analysis of deamidation kinetics, all protein samples were incubated at 25 °C in 75 mM sodium phosphate buffer, 100 mM NaCl (pH 7.3) with protein concentrations of 1.2 and 1.5 mg mL^–1^ for GII.4 Saga P-domains (Figures 2 and 3, respectively) and 1.6 mg mL^–1^ for all GII.4 VA387 P-domains. Samples were diluted 1:10 in 20 mM sodium acetate buffer (pH 4.9) immediately before IEX runs. Protein species were quantified by integration of the UV absorption at 214 nm using Unicorn v.7 software (Cytiva). N373 half-life times *t*_1/2_ were determined by fitting the relative amounts of respective N/N proteins against a two-parameter exponential decay model using Matlab 2020a (MathWorks).

### Determination of Deamidation Rate Constants

The following chemical equations were used to model deamidation of P-domain dimers. NN, iDN, and iDiD refer to dimeric proteins with different deamidation status that are composed of monomers N or iD.
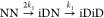
1
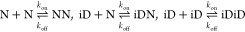
2
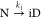
3The following ordinary differential equations describe the kinetics of this system of coupled reactions (adapted from ref (30)):
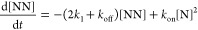
4
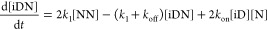
5
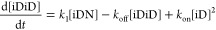
6
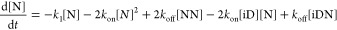
7
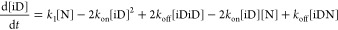
8

This system of ordinary differential equations was solved numerically using the previously determined^31^ dissociation rate constant *k*_off_ 1.51 × 10^–6^ s^–1^ and in-house Python (v2.7) scripts. Initial concentration values at *t* = 0 s were set to a relative concentration of 1 for the isolated starting species NN or iDN and zero for all the other species. The deamidation reaction rate constant *k*_1_ was varied from 0.01 to 10 × 10^–6^ s^–1^, and squared residuals were calculated between experimental and simulated data. *k*_1_ was determined by a least-squares approach. Monomer concentrations [N] and [iD] remain negligibly small for *k*_on_ = 10^3^–10 ^6^ M^–1^ s^–1^; i.e., the equilibrium lies almost completely on the side of the dimers in an affinity range of 1.5 nM to 1.5 pM. Accordingly, varying *k*_on_ in the given range has no discernible effect on the solution, and residuals of the curve fitting do not form a narrow minimum (Figure S1). Thus, *k*_on_ was set arbitrarily to 10^5^ M^–1^ s^–1^ as this is the order of magnitude recently determined for the homologous murine NoV protruding domain.^31^

### Protein NMR Spectroscopy

Protein NMR samples were prepared with a volume of 160 μL in 3 mm NMR tubes (Bruker). Spectra were acquired at 298 K on a 600 MHz Bruker Avance III HD NMR spectrometer with a TCI cryogenic probe unless stated otherwise. NMR sample conditions are given in Table S1. Methyl-α-l-fucopyranoside was purchased from Carbosynth. Spectra were processed using TopSpin v3.6 (Bruker) and analyzed using CcpNmr v2.4.^32^ Euclidean chemical shift perturbations were quantified and used for global fitting of dissociation constants *K*_D_ as described elsewhere.^21^

### Peptide Synthesis

The peptides were synthesized on an automatic peptide synthesizer (Syro I, Biotage) by using a Rink-amide resin and Fmoc chemistry. The Fmoc deprotection was carried out with 25% piperidine in DMF/NMP (70:30, v/v) for 3 min and 12.5% piperidine in DMF/NMP (70:30, v/v) for 12 min. The couplings were accomplished with the mixture Fmoc-AA-OH/HOBt/HBTU/DIPEA (5:5:4.8:10 equiv) for 2 × 40 min. N-terminal acetylation was performed manually with acetic anhydride/DIPEA (10:10 equiv) in DMF for 30 min. The peptides were cleaved from the resin with TFA/H_2_O/TIA/EDT/TIS (90:1:3:3:3; *V*_tot_ = 1 mL) for about 3 h, precipitated by ice-cold diethyl ether, and recovered by centrifugation at 4 °C for 5 min. The homogeneity and identity of the lyophilized peptides were assessed by analytical HPLC (Thermo Fisher Scientific) and MALDI-TOF-MS (Bruker Daltonics) (Figures S20 and S21 and Table S7).

### Peptide NMR Spectroscopy and Data Analysis

Samples were measured on a 600 MHz Bruker Avance III HD spectrometer equipped with a ^2^H/^13^C/^15^N/^31^P quadruple-resonance probe at 298 K. Volumes of 500 μL in standard 5 mm NMR tubes (Armar) were used. Standard 2D [^1^H,^1^H]-TOCSY, [^1^H,^1^H]-COSY, [^1^H,^1^H]-ROESY, [^1^H,^13^C]-HSQC, [^1^H,^13^C]-HMBC, and [^1^H,^15^N]-HSQC experiments were recorded at natural abundance. Spectra were processed with Topspin 3.2 (Bruker Biospin), referenced to 2,2-dimethyl-2-silapentane-5-sulfonic acid (DSS), and further analyzed in Sparky (T. D. Goddard and D. G. Kneller, SPARKY 3, University of California, San Francisco, CA). ^1^H and ^13^C chemical shift assignments of the original peptides and the species after deamidation are found in Tables S3 and S4.

For curve fitting of the decay of original Asn signals, Origin (MicroCal) was used. The signal intensities of Hα–Hβ correlations of Asn residues within [^1^H,^1^H]-TOCSY spectra as a function of incubation time were fitted with an exponential decay function using in-house Python (v2.7) scripts.

### Molecular Dynamics Studies

Theoretical conformational sampling was achieved using full-atomistic equilibrium molecular dynamics. Data were collected from five individual trajectory replicas of 1 μs length each. The trajectories were calculated using GROMACS 5.1.5 and GROMACS 2018.3,^33,34^ employing CHARMM36m force field parameters.^35^ Modeling of the initial systems was attained with CHARMM-GUI^36−38^ using the X-ray structures from PDB-ID 4OOX (SAGA) or PDB-ID 2OBT (VA387), TIP3P water,^39^ and 0.15 M NaCl ionization in a cubic box. The systems were minimized with the steepest descent method and briefly equilibrated for at least 0.1 ns in the NVT ensemble. For subsequent NPT production sampling at 303.15 K, a Nosé–Hoover thermostat^40^ and Parrinello–Rahman coupling^41^ were employed. The simulation time step was 0.002 ps, and conformations were saved every 20 ps. For each protein, 5 trajectories of 1 μs length each were simulated. We note that the VA387 simulations were performed later than the SAGA simulations, which gave us access to much faster GPU nodes. To take full advantage of the GPUs, we moved to a newer GROMACS version, with the consequence that a few updates were made to the simulation protocol and input parameters (see the SI).

Data analysis and visualization were carried out with VMD 1.9.3,^42^ GROMACS tools, and the Python packages NumPy,^43^ MDTraj,^44^ and MatPlotLib.^45^ Here, the side chain torsion angles of the Asn residues, as well as the distances from the Cγ atoms of Asn to the backbone nitrogen atoms of the subsequent amino acids, were monitored. φ is defined as torsion angle between C_i-1_-N_i_-CA_i_-C_i_, ψ between N_i_-CA_i_-C_i_-N_i+1_, *Χ*_1_ between N-CA-CB-CG, and *Χ*_2_ between CA-CB-CG-OD1. The free energy maps were constructed from the 2D probability densities as estimated by binning the data to 100 × 100 bins of 2π/100 widths. The relative free energies were computed as the negative natural logarithm of the probability density. Clustering of the 4D torsional angle space was achieved with the HDBSCAN^46^ method using an extended angle representation *z*(α) = [cos α, sin α]. More details are given in the SI. One of the 5 MD trajectories of the SAGA P-dimer has been used to generate conformers for ensemble docking in an earlier study.^47^

## Results

### Modeling of Simultaneous Deamidation of N373 and P-Dimer Dissociation

We have shown previously that the kinetics of deamidation of N373 of GII.4 Saga P-dimers can be studied using analytical cation exchange chromatography (IEX). Three peaks were observed in the IEX chromatograms, reflecting the three different charge states of the homodimeric P-domains: non-deamidated (N/N), partially deamidated (iD/N), and fully deamidated (iD/iD) (Figure 2a). Fitting a simple exponential function to the decaying intensities of the N/N peaks yielded an estimate for the half-life characterizing the spontaneous deamidation reaction.^12^ At that time, however, we were not aware that the deamidation reaction proceeds on the same time scale as the dissociation of P-dimers.^31^ Therefore, a more realistic kinetic analysis of deamidation must consider concurrent P-dimer dissociation as described below.

As the kinetic model applied (Figure 2b) assumes formation of isoaspartate as the only product of N373 deamidation, we performed additional experiments to exclude the formation of aspartate. We synthesized [*U*-^2^H,^15^N]-labeled P-domains carrying the N373D mutation and acquired ^1^H,^15^N TROSY HSQC NMR fingerprint spectra that were compared to corresponding spectra of an aged, fully converted native P-dimer sample (Figure 1b). No signals characteristic of the N373D mutant were detected in spectra of the converted P-dimer sample, proving that only an isoaspartate is formed upon deamidation of N373.

Incubating non-deamidated N/N Saga P-dimer samples at 25 °C showed the formation of asymmetric iD/N and fully deamidated iD/iD P-dimers (Figure 2c). However, global fitting of N/N, iD/N, and iD/iD IEX peak intensities to the simplest model of two consecutive, irreversible reactions poorly matched the experimental data. Particularly, during incubation experiments starting with purified iD/N P-dimers, a noticeable fraction of non-deamidated N/N P-dimers reemerged (Figure 2d). However, Asn deamidation is an irreversible reaction. Therefore, the appearance of N/N P-dimers can only be explained by dissociation of iD/N P-dimers into monomers, with subsequent reassembly generating N/N as well as iD/iD dimers (Figure 2b). Therefore, we incorporated the dissociation of P-dimers into monomers and subsequent stochastic reassembly into the different dimer species into the model, resulting in a system of differential rate equations (eqs 4–8). The dimer dissociation rate constant *k*_off_ is available from our previous study into the dimer–monomer equilibrium of stable point mutants of Saga P-dimers,^31^ specifying *k*_off_ as 1.5 × 10^–6^ s^–1^. The solution of the system of differential equations is almost independent of the association rate constant *k*_on_, as in this system monomer concentrations remain negligibly small (corresponding to a dimerization dissociation constant in the nM–pM range). This leaves the deamidation rate constant *k*_1_ as the only parameter to be fitted. Solving the differential equations numerically and varying *k*_1_ allows least-squares fitting (Figure S1) and yielded excellent curve fits (Figure 2c) with *k*_1_ being 4.5 × 10^–7^ s^–1^ (or 0.04 day^–1^). Two experimental data sets, starting either with purified N/N or with iD/N dimers, have been analyzed independently to validate the proposed model of deamidation accompanied by P-dimer dissociation and reassembly. N/N and iD/N data sets yielded almost identical results (Figure 2c,d). Notably, deamidation rates also strongly depend on buffer conditions. The rate constants and half-life times were determined at pH 7.3 at 25 °C, but acidic buffers can extend the N373 half-life to over 100 days at 25 °C (Figure S2).

### Comparison of Different GII.4 NoV Strains

Selection pressure of the host immune system causes considerable sequence variation within the outward facing parts of the HuNoV capsid protein VP1,^48^ including the loop containing N373. High conservation of N373 among GII.4 strains suggests a functional advantage of Asn in this position. Therefore, we investigated the impact of sequence variation in neighboring positions on the deamidation behavior of a natural GII.4 NoV variant, the VA387 strain. P-domains of the Saga and the VA387 strains are remarkably similar in terms of sequence (90% identity) and 3D structure (0.4 Å RMSD) (Figure 3a and Figure S3). However, two amino acid point mutations R297H and E372N are close to the critical position 373, which allowed us to study deamidation in the context of two naturally occurring protein homologues. Upon aging of [*U*-^2^H,^15^N]-labeled samples of VA387 P-domains, we identified the same changes in the HSQC cross peak patterns characteristic for N373 deamidation in Saga P-domains, demonstrating that site-specific deamidation is conserved among the two strains (Figures S3 and S4). Likewise, for both P-dimers, only isoAsp (iD) was detected as the product of deamidation (Scheme 1, Figure 1, and Figures S5 and S6). Using IEX, we investigated the half-life of N373 in VA387 N/N P-dimers. For reasons of improved protein stability, these experiments were performed at 25 °C instead of 37 °C used in our previous study. Interestingly, *t*_1/2_ in VA387 is 27 days, significantly longer than the 9 days we observed for the Saga strain (Figure 3b).

To probe potential differences in local conformations of the loop that could account for this divergence, we determined the dissociation constant *K*_D_ for binding of methyl α-l-fucopyranoside to VA387 P-dimers. It is known that D374 is critical for binding to l-fucose-containing glycans, and therefore, such conformational changes may reflect on binding affinities. Titration of [*U*- ^2^H,^15^N]-labeled VA387 N/N P-dimers with methyl α-l-fucopyranoside and observation of chemical shift perturbations (CSPs) in ^1^H,^15^N TROSY HSQC spectra yielded a dissociation constant *K*_D_ of 21 mM (Figure S8), almost identical to the value previously determined for GII.4 Saga P-dimers.^12^ This suggests that at least fucose recognition is similar between both strains. Next, we created point mutants to further examine the observed difference in deamidation rates. There are two amino acids in spatial proximity to N373 that differ in the VA387 and Saga strain. In VA387 P-domains, the *i* – 1 residue E372 is exchanged for another Asn, N372, and position 297 in a neighboring loop contains a His residue, H297, instead of an Arg297 in the Saga strain. We mutated both positions in VA387 P-dimers into their respective GII.4 Saga counterparts and then monitored deamidation. Both mutations substantially increased deamidation rates. Surprisingly, the H297R mutant alone almost restored the fast deamidation kinetics of the Saga strain, clearly indicating that deamidation of N373 is controlled by an interaction with a neighboring surface loop and not by the sequence. As expected, the behavior of the H297R/N372E double mutant of VA387 closely resembles that of the Saga wild type protein.

### Deamidation of P-Domain Model Peptides Is Orders of Magnitude Slower

**Figure 4:**
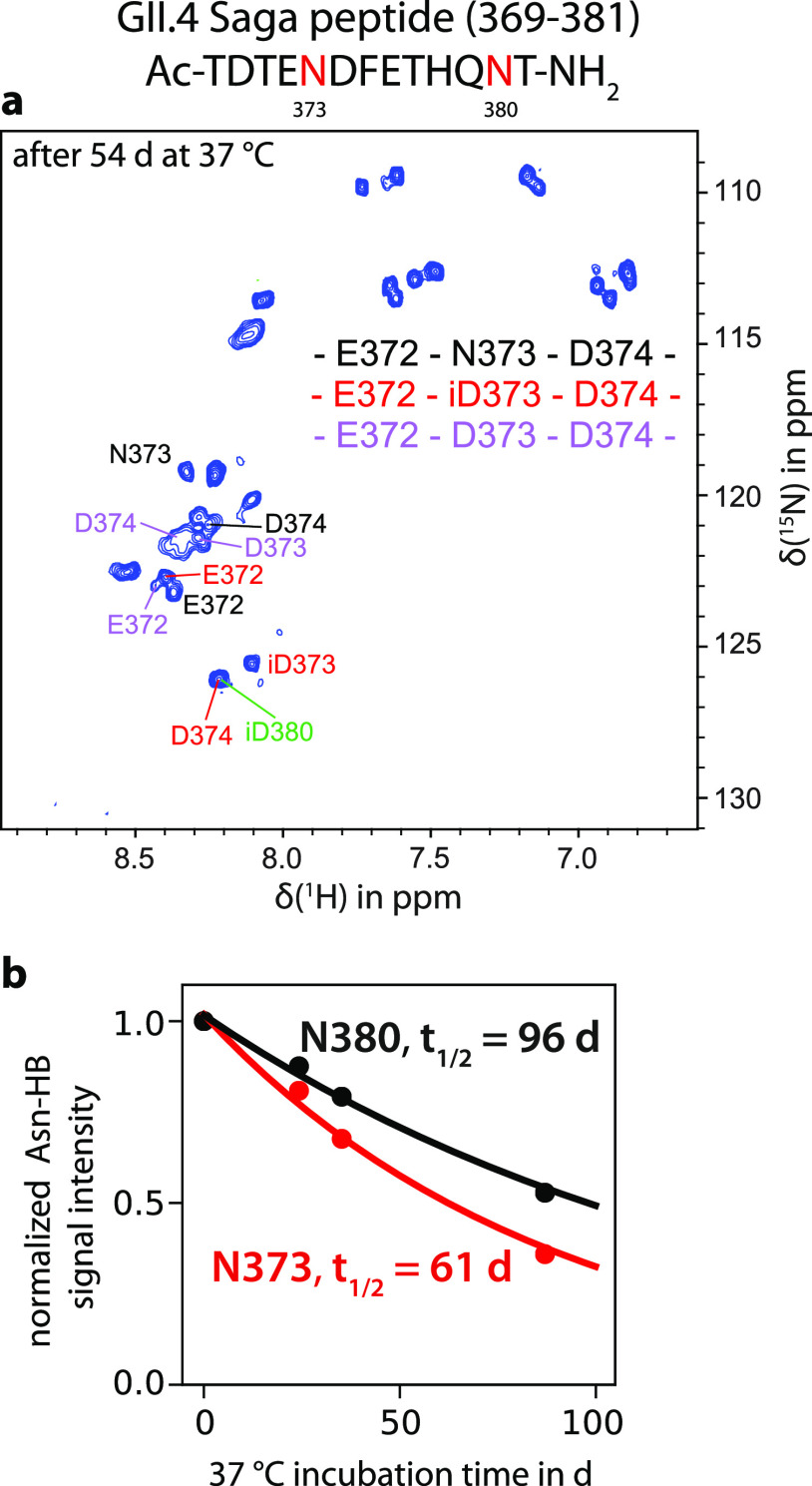
Deamidation of a 13-mer peptide mirroring the amino acid sequences of the deamidating loop of GII.4 Saga P-domain proteins. (a) ^15^N HSQC NMR spectra of the model peptide after incubation at 37 °C for 54 days reveal that deamidation is not exclusive for N373 but can be observed for N380 as well. Additionally, both reaction products isoAsp and Asp can be detected with a ratio of 4:1 (red and violet sequences, respectively). Amino acids have been numbered according to their position in the full-length protein for clarity. (b) NMR signal intensities from characteristic HB signals reporting for the respective Asn were obtained from TOCSY spectra (Figure S10) and were used to fit an exponential decay model to yield Asn half-lives *t*_1/2_.

To dissect a possible influence of the amino acid sequence on the deamidation rate of N373 from structural through-space effects, we synthesized 13-mer model peptides for both GII.4 Saga and VA387 P-domains. The peptides consist of the entire sequence of the loop that contains the deamidation site. Notably, these peptides contain multiple Asn residues allowing us to probe the selectivity for deamidation of N373 as well as the corresponding reaction kinetics (Figure 4). To this end, we monitored 2D NMR spectra of the peptides under the same buffer conditions as applied to the P-dimers. Signals for several new species emerged during incubation of the peptide samples at 37 °C. In contrast to the P-dimers, all Asn residues in the peptides deamidated over time, and both isoAsp and Asp reaction products were detected with a ratio of ca. 4:1. Of note, the ^15^N chemical shifts of the backbone amide of the formed isoAsp and of the *i* + 1 residue were surprisingly high at 125–126 ppm. This observation was also made for the corresponding signals of isoAsp373 (iD373) and Asp374 (D374) in Saga P-domains [both: δ(^15^N) = 125.8 ppm^12^]. Apparently, ^15^N chemical shifts of isoAsp were mostly independent of the conformational context. This is supported by the previous observation of a random coil chemical shift in an isoAsp-containing model peptide at 124.2 ppm (BMRB entry 50601). Therefore, appearance of a signal in this spectral region may in general serve as an indicator of deamidation.

The decrease in NMR signal intensities of the different Asn residues over time allowed us to estimate Asn half-life times for the model peptides (Figure 4b and Figure S9). We found that, even at higher temperatures, Asn deamidation at the position corresponding to N373 was significantly slower than in the P-dimer, i.e., 61 days at 37 °C for the Saga peptide compared to 1.6 days for the protein. The Asn residue at the position corresponding to N380 deamidated even slower with a half-life of 100 days. As deamidation in the model peptide is neither fast nor exclusive for N373, we conclude that fast deamidation of N373 of Saga P-dimers is primarily caused by conformational effects.

### Standard Descriptors Do Not Predict N373 Deamidation

**Figure 5:**
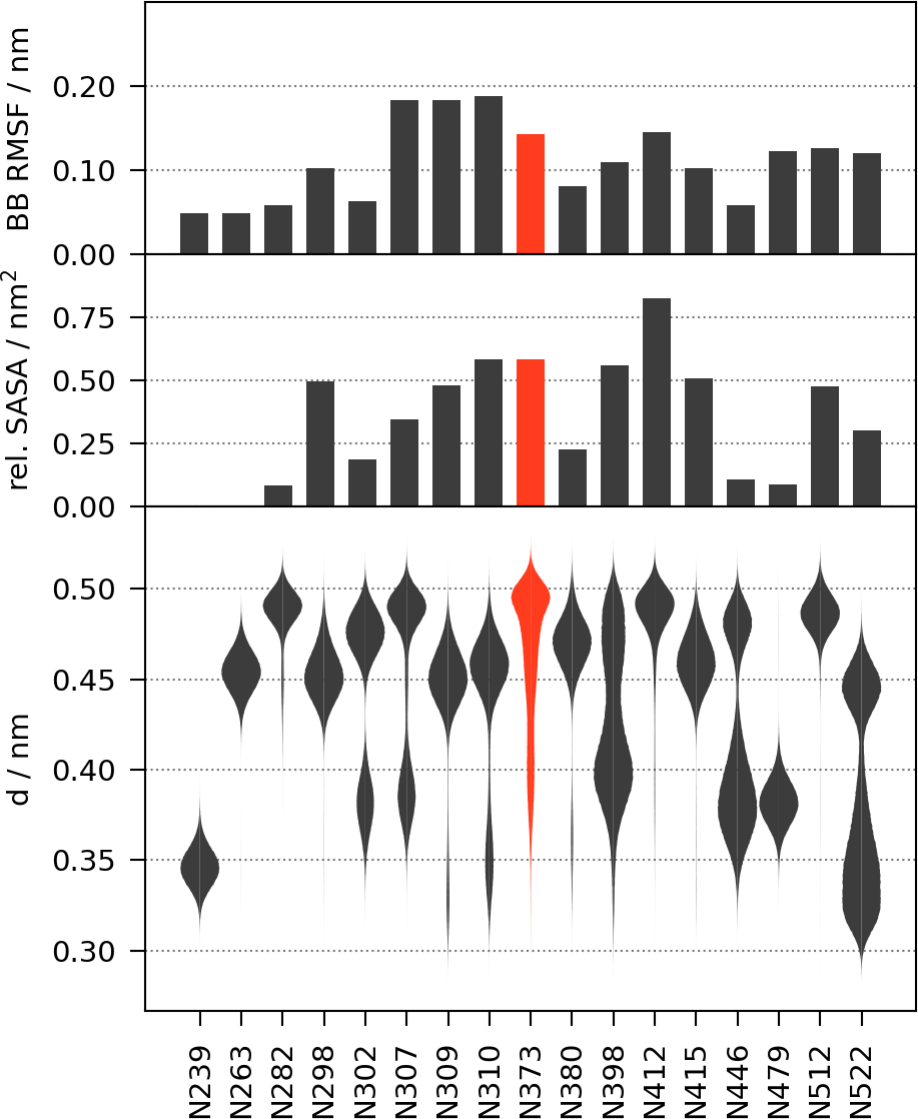
Comparison of standard deamidation descriptors for all Asn residues in the Saga P-domain dimer computed from the MD simulation. N373 is highlighted in red. Panels show, from top to bottom, the average backbone RMSF, the average relative solvent accessibility (0, buried; −1, maximum solvent accessible), and the probability density functions of the C^γ^_*i*_–N_*i*+1_ attack distances in violin representation. The density functions are estimated using 200 bins and scaled by the maximum probability (areas are not equal to 1). The averages are computed as the means of the replica trajectory means. The density was computed from the pooled data of all trajectories.

Attempts to identify factors explaining fast and selective deamidation of N373 of GII.4 Saga P-dimers using sequence- and crystal structure-based methods provided inconclusive results. Therefore, we conducted extensive, multi-microsecond molecular dynamics (MD) simulations extending the analysis to an entire conformational ensemble of P-dimers. From the MD trajectories, we calculated distributions of well-established descriptors for Asn deamidation such as backbone root-mean-square fluctuations (RMSF) and solvent accessible surface areas (SASA). As expected, N373 was among the more solvent-exposed and flexible Asn residues. However, a comparison with other, non-deamidating Asn revealed no outstanding properties of N373 that would explain its atypical deamidation behavior (Figure 5). Similarly, the sampling frequency of conformations providing short attack distances (*d*(C^γ^_*i*_–N_*i*+1_) < 0.4 nm) required for deamidation would classify several Asn residues as “reactive”—even those that are stable on the experimental time scale of weeks to months. Not every conformational arrangement resulting in a short distance between nucleophile and electrophile is necessarily favorable in terms of overlapping frontier molecular orbitals. Accordingly, we extended our analysis to include the nucleophile approach trajectory angles *α*_BD_ (Bürgi–Dunitz)^49,50^ and *α*_FL_ (Flippin–Lodge)^51,52^ as descriptors of the nucleophilic attack trajectory. Computing joint probabilities to identify near-attack conformations in which all the geometric requirements (*d* < 0.4 nm, 45° < *α*_BD_ < 135°, −45° < *α*_FL_ < 45°) are satisfied simultaneously, ranked N373 only in the midtier of all P-domain Asn residues (Table S5).

### An Unusual Asn Backbone Conformation Might Explain Reactivity

**Figure 6:**
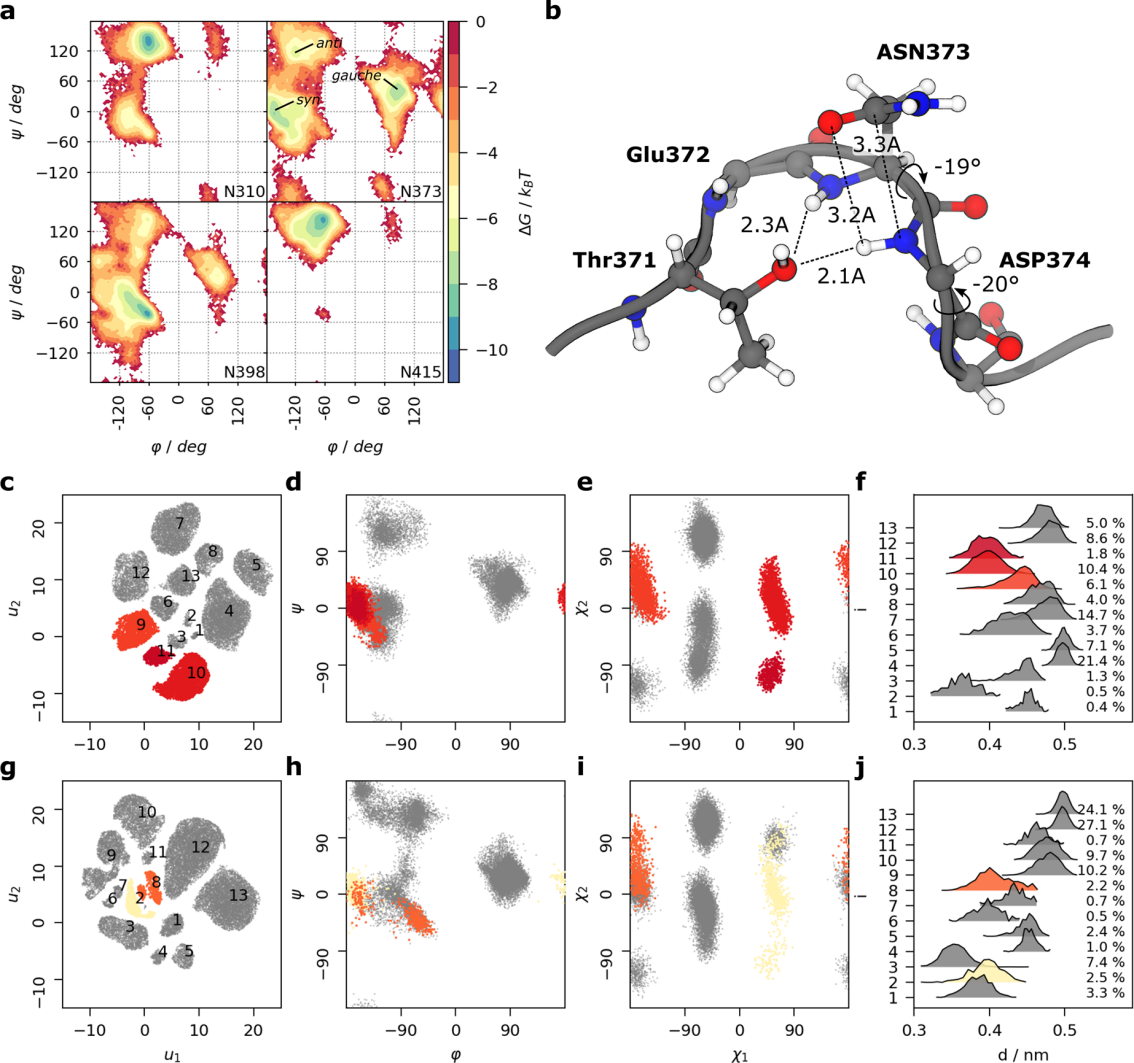
Illustration of the population of an unusual *syn*-backbone conformation of N373 in Saga and VA387 P-dimers. (a) Free energy maps of backbone torsion angles φ and ψ for N373 and representative Asn residues of the GII.4 Saga P-dimer. The complete set of maps for all Asn residues is shown in Figure S11. The data were pooled from 5 individual replica simulations of 1 μs sampling time each, as well as both monomeric chains. The predominating backbone conformations of N373 are annotated. (b) Corresponding snapshot from the MD simulation depicting a double *syn* conformation. (c–j) Comparative conformational clustering of the torsion angles of N373 of GII.4 Saga (c–f) and of VA387 (g–j). (c, g) UMAP 2D embedding of the torsion and side chain angles colored by identified clusters. Cluster IDs are annotated. (d, h) Backbone torsion angles φ and ψ. (e, i) Side chain torsion angles *χ*_1_ and *χ*_2_. (f, j) Attack distance distributions in each cluster, showing how they contribute to the total conformational space. The histograms are scaled to have equal areas. Conformational clusters that are reactive according to the backbone-distortion hypothesis are colored in shades of red (Saga) and yellow (VA387). Other clusters are colored gray. Fully colored versions of this figure are given in Figures S14 and S15.

Inspection of the backbone torsion free energy landscape of all Saga P-domain Asn residues revealed a unique feature of the deamidating N373. Most of the Asn residues populate one or more of the three distinct energy minima that belong to the well-established β-sheet-like (φ, ψ ≈ −120°, 120°), α-helical (φ, ψ ≈ −90°, 0°), and left-handed α-helical (φ, ψ ≈ 60°, 45°) conformations (Figure 6a and Figure S11). N373 stands out by a much shallower free energy landscape as reflected by minima for N373 being ca. 4 *k*_B_*T* higher compared to other asparagine residues (Figure 6a and Figure S11). Importantly, a unique, highly populated energy minimum (φ, ψ ≈ −180°/0°) is exclusively accessible to N373 and corresponds to an unusual backbone *syn* conformation, in which the nitrogens N_*i*_ and N_*i*+1_ are nearly eclipsed. To better understand which areas in the four-dimensional space of backbone and side chain torsion angles φ, ψ, *χ*_1_, and *χ*_2_ (Figures S11 and S12) are associated with potential Asn reactivity, we employed conformational clustering (Figure 6c–e). Three clusters are sampling the backbone *syn* conformation and (partially) allow for short C^γ^_*i*_–N_*i*+1_ attack distances (Figure 6f). We hypothesize that access to the associated conformations is crucial for Asn reactivity. Interestingly, the hydrogen bond between T371 and D374 stabilizes this *syn* conformation creating a type II′ ST turn motif.^53^ The observed simultaneous hydrogen bonding between T371–D374 and T371–N373 (Figure 6b) is only possible with N373 residing in a *syn* conformation. This, in fact, leads to two consecutive *syn*-backbone orientations of residues N373 and D374.

We extended our MD analysis to P-domains of the more slowly deamidating strain VA387 (Figures S13–S15). Again, among all Asn residues only N373 can access the potentially reactive *syn* conformation (Figure S15). However, in VA387 this conformation is adopted less frequently than in the Saga strain. In VA387, reactive conformational clusters were sampled in only 5% of frames compared to 18% observed in the Saga strain (Figures 6, Figures S16 and S17). We analyzed the MD trajectories for Saga and for VA387 P-dimers with respect to the stability of the ST turn motif by determining the occupancy of the T371–N373 and T371–D374 hydrogen bonds during the simulations (Figures S18 and S19). For Saga, the two hydrogen bonds are present at 40% (T371–N373) and 50% (T371–D374) of the time, indicating a transient, metastable secondary structural motif. In VA387, these occupancies are significantly decreased to 25% (T371–N373) and 20% (T371–D374). In line with these observations, near-attack conformations with favorable geometric arrangement of nucleophile and electrophile are sampled 2.6 times less frequently in the VA387 strain (Table S6).

## Discussion

The analysis of our MD simulations suggests that, in addition to conformations providing a short attack distance and optimal attack trajectory, the nucleophilicity of the attacking nitrogen seems to be crucial. N373 in GII.4 norovirus P-dimers populates a shallow minimum in the free energy landscape around φ, ψ ≈ −180°, 0°. The associated adoption of an unusual *syn*-backbone conformation seems to be linked to the fast deamidation and subsequent exclusive formation of an isoaspartate residue. A comparison of deamidation rates of two structurally almost identical P-dimers of different GII.4 strains, Saga and VA387, allows further insight into conformational factors controlling the deamidation rate. The deamidation rate of Saga P-dimers is found to be larger by a factor of 3. Most of this effect is shown to be due to a single point mutation, H297R, in a loop neighboring the loop containing the critical N373 (cf. Figure 3). This suggests that this neighboring loop modulates the stability of the *syn*-backbone conformation of N373 and thus the deamidation rate. In excellent agreement with this experimental finding, the MD simulations predict that the favorable attack geometry with the *syn* conformation is sampled more frequently in Saga than in VA387 P-dimers. In the following, we put these new findings into perspective by discussing why existing methods to predict deamidation sites failed to rationalize our findings.

At the simplest level one may use pentapeptide-derived sequence rules, predicting short Asn-deamidation half-life times for Asn followed by a Gly residue.^10^ However, neither of the two Asn–Gly dipeptide sequences in the Saga P-domain shows any signs of deamidation in the time window of three months. The same study shows that an Asp residue in the *i* + 1 position leads to half-lives between 30 and 40 days in peptides, which is in contrast to the fast deamidation of Asn373 as part of the sequence Glu372–Asn373–Asp374 of the Saga HuNoV P-domain. This illustrates that prediction methods solely based on the sequence are not sufficient to explain the experimental observations. More sophisticated sequence-based algorithms^54^ exist but also fail to identify Asn373 as being prone to fast deamidation. The experimental half-lives that we determined for longer synthetic peptides matching the amino acid sequence of the loop containing N373 support these data (cf. Figure 4), and half-lives in the range of months are measured.

Prediction of Asn deamidation based on 3D structure models should provide a better match with experimental data. Therefore, several studies have addressed the question of which structure-associated descriptors may be relevant for the accurate prediction of deamidation sites. Our initial analysis of potential causes for fast N373 deamidation has been limited to available crystal structure models, in some cases suffering from poor electron density at critical positions. Accordingly, we extended our study to the entire conformational ensemble of the protein sampled in several μs of MD simulations. However, none of the established descriptors revealed any unique properties of Asn373 that would qualify it for fast deamidation. We argue that this might be an indication of poor representation of very fast deamidation events in previous studies. For example, data sets contained only 3% of comparably fast reactions in ref (27) and 0% in refs (10 and 24).

**Scheme 2:**
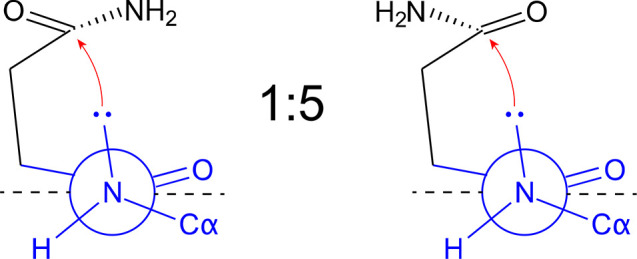
Schematic Representation of the Backbone Twisting Associated with Pyramidalization of the Nitrogen and Carbonyl Atoms At the same time, the nitrogen undergoes a rehybridization from sp^2^ to sp^3^ increasing its nucleophilicity. Shown are two reactive rotamers of the side chain of N373 of Saga P-dimers characterized by the χ_1_ and χ_2_ values given in Figure 6e (red clusters). The indicated ratio refers to the relative cluster populations of clusters 10 and 11 given in Figure 6f.

We finally asked the question why the unusual *syn*-backbone conformation of N373 with φ, ψ ≈ −180°, 0° is associated with fast deamidation. Interestingly, population in this unusual area of the Ramachandran plot has been observed before for a residue preceding a scissile bond.^55^ It seems to be linked to a special type of backbone distortion, namely, amide twisting, which is associated with a *syn*-backbone conformation. We can profit from the highly advanced hybrid density functional theory calculations of the model tripeptide Gly–Gly−ε-Lys by Strieter and Andrew.^55^ These authors found that a twisting around the (iso)peptide bond leads to a pyramidalization of the carbonyl, an increased sp^2^ character, and thus higher electrophilicity, which is central to their discussed mechanism of isopeptide-bond cleavage. Interestingly, and more important for deamidation, is that peptide bond twisting leads to an even larger pyramidalization of the nitrogen, which is accompanied by rehybridization from sp^2^ to sp^3^ and thus generating a free electron pair at the nitrogen. This electron pair substantially increases nucleophilicity of the nitrogen and gives it a clear direction. Strikingly, this peptide bond twisting is reflected by unusual backbone dihedral angles as seen in Ramachandran plots around φ ≈ −170° to −70°, and ψ ≈ −40° to 40°. This is exactly the region that we observe to be populated by N373 (Figure 6a). This conformation is characterized by a *syn* conformation in which the backbone N–H vectors of N373 and D374 are pointing toward the side chain oxygen of T371. Strieter and Andrew further observe in their calculations that the closer ψ is to 0°, the stronger the sp^2^ character of the carbonyl and the stronger the sp^3^ hybridization of the nitrogen. In the GII.4 Saga P-dimer the φ angle of N373/D374 of the highly populated *syn* conformation (Figure 6a) is indeed centered around 0°. This suggests that the nitrogen of D374 has partial sp^3^ hybridization, and the free electron pair oriented toward the Asn373 side chain carbonyl can perform a nucleophilic attack (Scheme 2). Amide twisting was also observed in an oligosaccharyltransferase in which an asparagine side chain is activated so that the nitrogen acts as nucleophile.^56,57^

In the P-dimers, we only observe isoAsp but no Asp as product of deamidation. That could reflect a preference of the reaction of the succinimide intermediate. Interestingly, the observed *syn* conformation and the associated backbone twisting increase not only the nucleophilicity of the nitrogen but also the electrophilicity of the backbone-carbonyl, making this site very susceptible to attack by water and yielding only isoAsp as a product. Instead of passing through a succinimide intermediate, a concerted mechanism, i.e., simultaneous nucleophilic attack of the backbone D374 nitrogen and of the water, should also be considered.

This mechanism also allows rationalization of the finding that the side chain of residue 297 has an influence on the deamidation rate (Figure 3). An H297R point mutant of the VA387 protein as found in GII.4 Saga deamidates faster than the wild type VA387 protein. Residue 297 undergoes significant interactions with D370/T371 (Figures S18 and S19) which is part of the type II′ ST turn (Figure 6b) and, therefore, might be one of the causes of strain around the backbone between D370 and D374. An Arg in this position needs more space and can potentially form π–π stacking interactions with the peptide bond D370/T371, which is reflected by higher R297–D370/T371 contact occupancies in the GII.4 Saga P-dimer relative to H297–D370/T371 in VA387. Position 372 is part of the strained type II′ ST turn and could also modulate the strain of the backbone.

We suggest that, in addition to a substantial population with a favorable attack geometry, nucleophilicity of the attacking nitrogen is an important factor making Asn residues susceptible for deamidation. We hypothesize that backbone distortion reflected by an unusual population around φ, ψ angles ≈ −170°, 0° leads to a twisting of the peptide bond, resulting in pyramidalization of the attacking nitrogen and thus increasing its nucleophilicity. Future prediction algorithms for sites of fast deamidation of Asn residues may profit from this finding.

## Supporting Information Available

Additional ion exchange chromatographs, 2D NMR spectra, chemical shift assignment tables, deamidation rate data, extensive analysis of the MD simulations, and supplementary methods (PDF)

## Accession Codes

The NCBI accession IDs are as follows: GII.4 Saga VP1, AB447457; and GII.4 VA387 VP1, AY038600. The corresponding links are https://www.ncbi.nlm.nih.gov/nuccore/AB447457 and https://www.ncbi.nlm.nih.gov/nuccore/AY038600.3/.

## Abbreviations

NoV: norovirus
HBGA: histo blood group antigen
TROSY: transverse relaxation-optimized spectroscopy
HSQC: heteronuclear single quantum coherence
IEX: ion exchange
CSPs: chemical shift perturbations
RSMF: root-mean-square fluctuations
SASA: solvent accessible surface area
