# Supplementary material for: Conformational Control of Fast Asparagine Deamidation in a Norovirus Capsid Protein
Source: Biochemistry. 2023 Feb 21;62(5):1032–43. doi: 10.1021/acs.biochem.2c00656 (PMC9996831; doi:10.1021/acs.biochem.2c00656)
Supplement: Supplementary file 1 — bi2c00656_si_001.pdf [file bi2c00656_si_001.pdf]

## Supporting Information for:

# Conformational control of fast asparagine deamidation in a norovirus capsid protein

Robert Creutznacher[†,‡], Eric Schulze-Niemand[†,§], Patrick König[‡], Vesna Stanojlovic[⊥], Alvaro Mallagaray[‡], Thomas Peters[‡,*], Matthias Stein[§,*], Mario Schubert[⊥,*]

[‡] *Institute of Chemistry and Metabolomics, University of Lübeck, Ratzeburger Allee 160, 23562 Lübeck, Germany,* [§] *Max Planck Institute for Dynamics of Complex Technical Systems, Molecular Simulations and Design Group, Sandtorstrasse 1, 39106 Magdeburg, Germany, and Department of Biosciences and Medical Biology, University of Salzburg, Hellbrunnerstrasse 34, 5020 Salzburg, Austria*

[†] R.C. and E.S.-N. contributed equally to this paper.

***Corresponding Authors**
Mario Schubert − email: mario.schubert@plus.ac.at
Matthias Stein – email: matthias.stein@mpi-magdeburg.mpg.de
Thomas Peters – email: thomas.peters@uni-luebeck.de

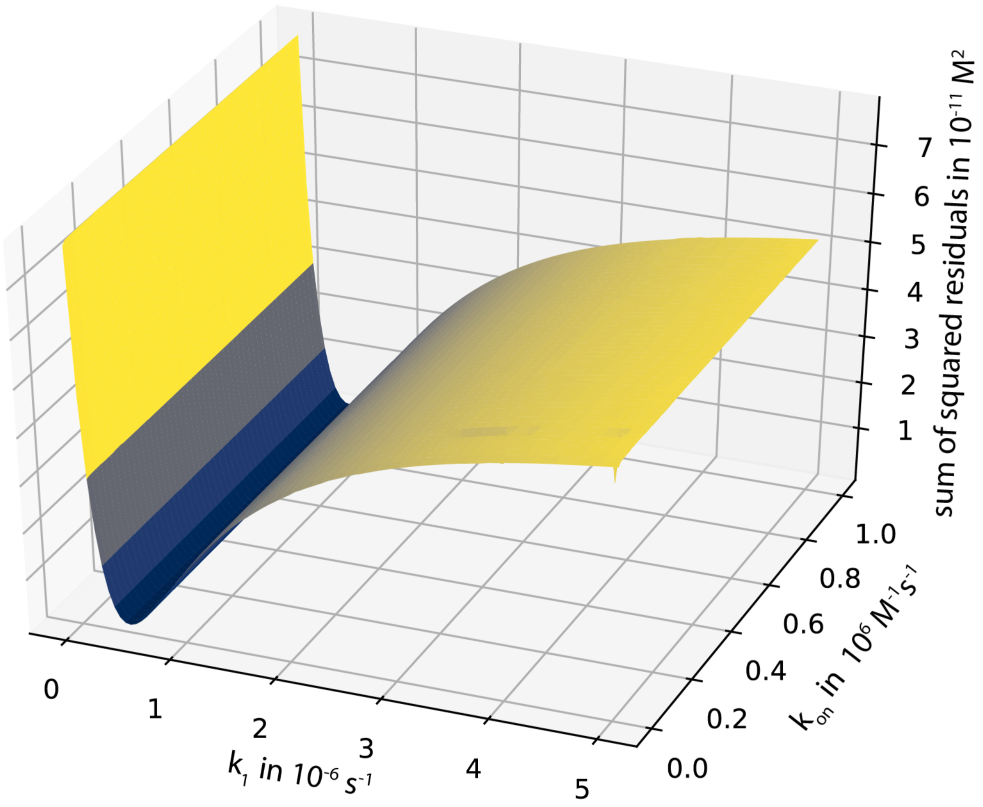


**Figure S1.** Residuals from fitting of the deamidation reaction rate in Fig. 2c. Fitting of experimental IEX data to the numerical solution of the system of differential equations describing simultaneous deamidation and dimer reassembly allows for determination of the deamidation rate $k_1$ but not of the association rate $k_{on}$. Independent of $k_{on}$, variation of $k_1$ leads to a narrow minimum in squared residual of the fit at $4.5\times10^{-7}$ $s^{-1}$. Accordingly, $k_{on}$ cannot be determined from curve fitting of the IEX data and its variation in the range $10^3$ – $10^6$ $M^{-1}$ $s^{-1}$ has no discernible effect on the solution.

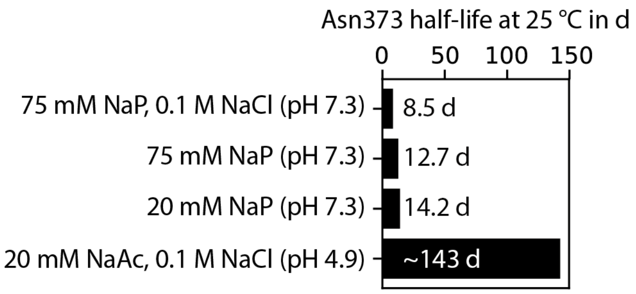


**Figure S2. Deamidation and dissociation rates depend on buffer composition.** Incubation experiments of GII.4 Saga P-dimers at 25 °C in different sodium phosphate (NaP) and sodium acetate (NaAc) buffers and subsequent IEX analysis reveal strong pH and ionic strength dependencies of the combined rate of deamidation and dissociation.

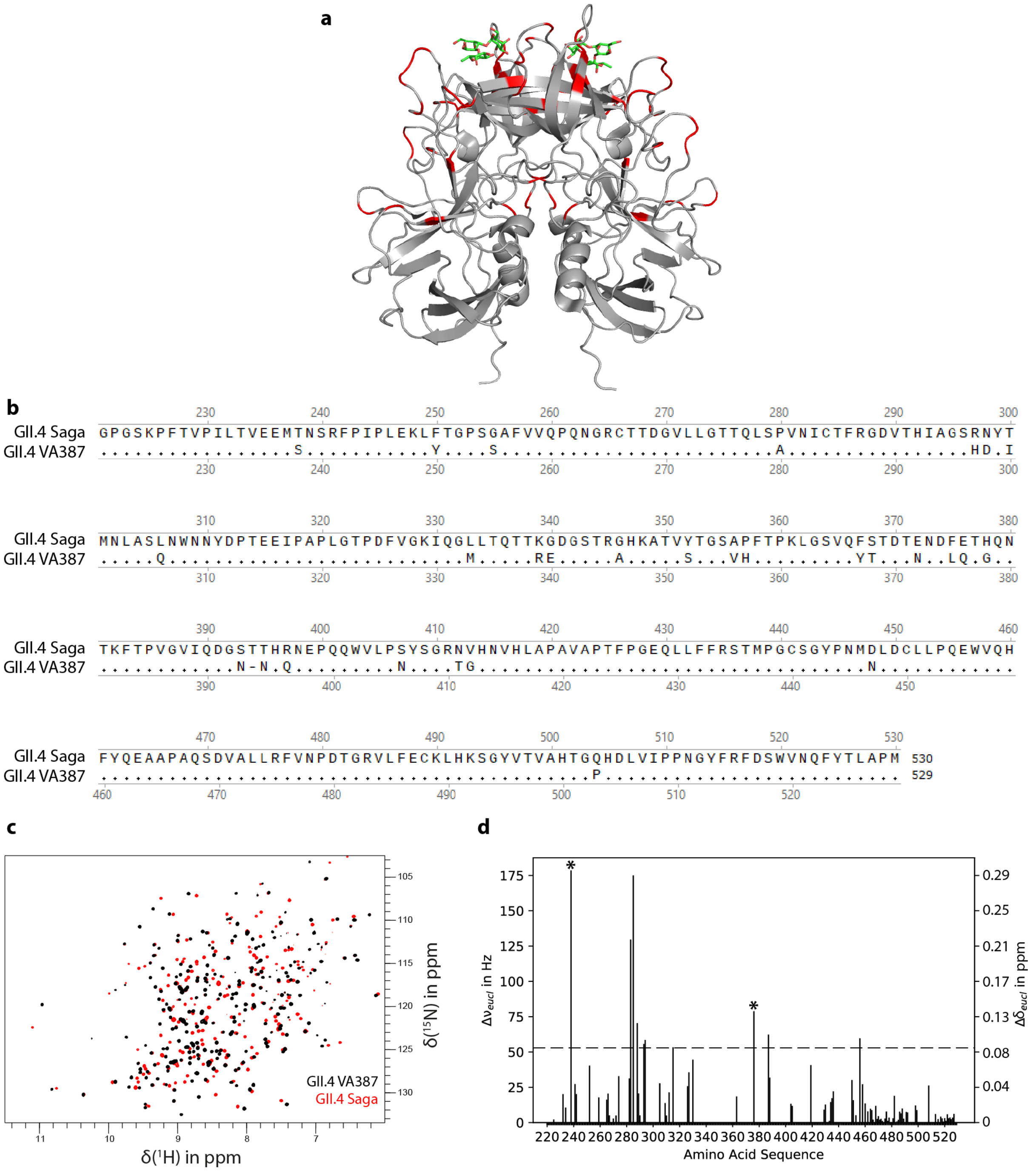


**Figure S3**. Comparison of GII.4 Saga and VA387 P-domains. (**a**) Amino acid sequence differences between both proteins were mapped on the crystal structure model of GII.4 Saga (pdb: 4x06). The blood group B trisaccharide – a representative HBGA – is shown in green. (**b**) A sequence alignment of both proteins shows 90% sequence identity. (**c**) TROSY HSQC spectra of both [$U$-$^{2}$H,$^{15}$N]-labeled P-domains in 75 mM sodium phosphate buffer, 100 mM NaCl, pH 7.3 (VA387: black, Saga: red). (**d**) Euclidean chemical shift differences between GII.4 Saga and VA387 P-domains. Signal assignments from the GII.4 Saga P-domain [1] have been transferred to the VA387 strain using the superimposed spectra (c) and the sequence alignment. Generally, an assignment transfer was only possible in isolated spectral regions and for signals corresponding to conserved amino acids (89/309 signals, cf. Table S2). Accordingly, assignment coverage is highest in the conserved C-terminal region of the protein and most observable chemical shift differences are small. Large chemical shift differences (threshold: mean + one standard deviation, dashed line) can be found for amino acids that are not conserved (asterisks, e.g. T238S) or that are close by to such regions (e.g. I283 and T285 neighboring the P280A substitution).

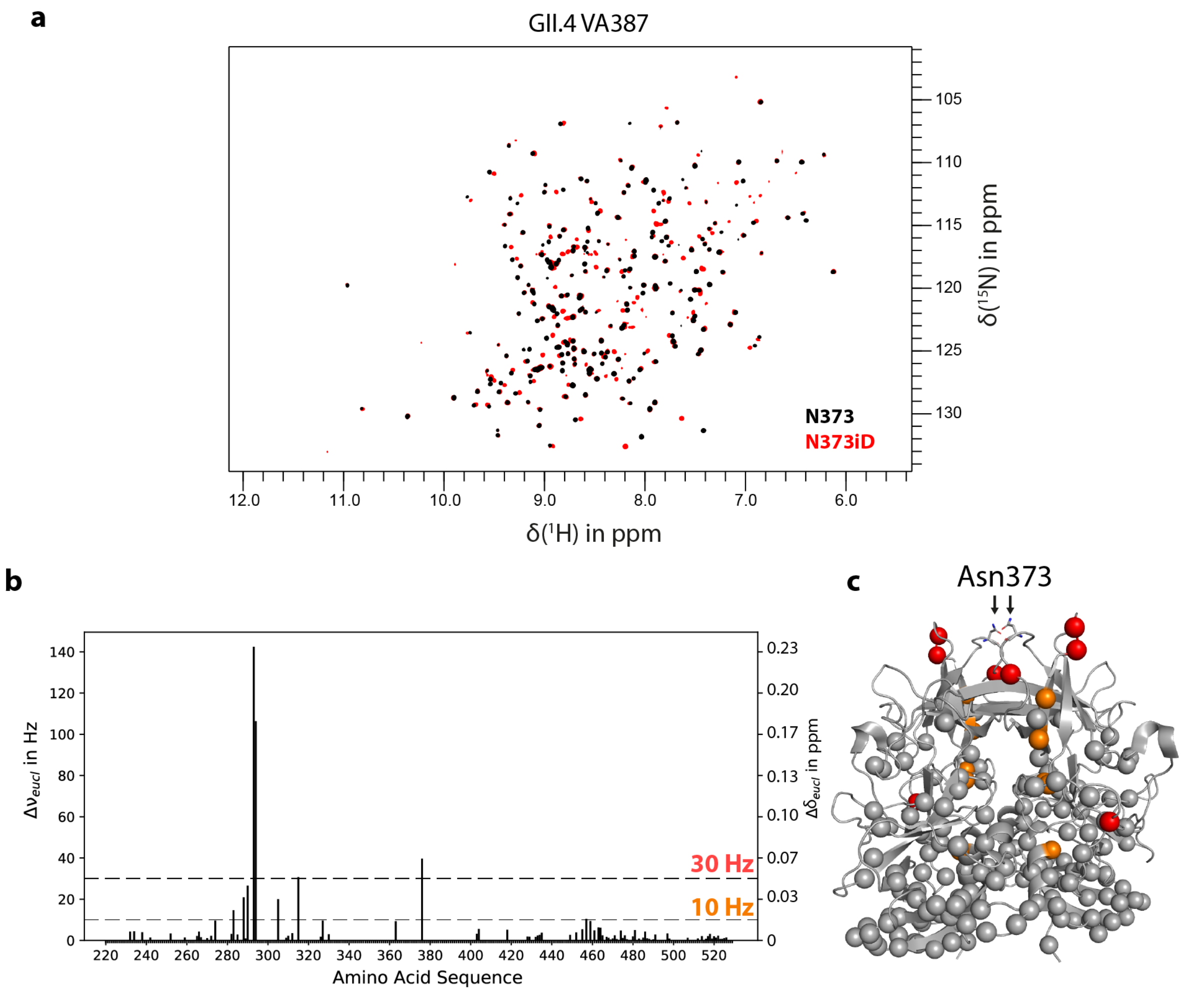


**Figure S4**. Chemical shift differences between non-deamidated GII.4 VA387 P-domains (N373) and fully deamidated ones (N373iD). Pure samples of the respective major protein species were obtained by preparative ion exchange chromatography (cf. Figure S7). (**a**) TROSY HSQC spectra of N373 (black) and N373iD (red) [*U*- $^{2}$H, $^{15}$N]-labeled P-domains were acquired in 75 mM sodium phosphate buffer, 100 mM NaCl, pH 7.3. Signal assignments for the N373 form were obtained for some signals following the procedure outlined in Figure S1. (**b, c**) Euclidean chemical shift differences between N373 and N373iD proteins have been obtained for 85 amino acids. Large differences can be found in the outward facing part of the protein, close to the deamidation site N373, closely resembling those observed for GII.4 Saga (cf. Figure 1, e.g. I293 and A294 in the bottom right corner of the spectrum).

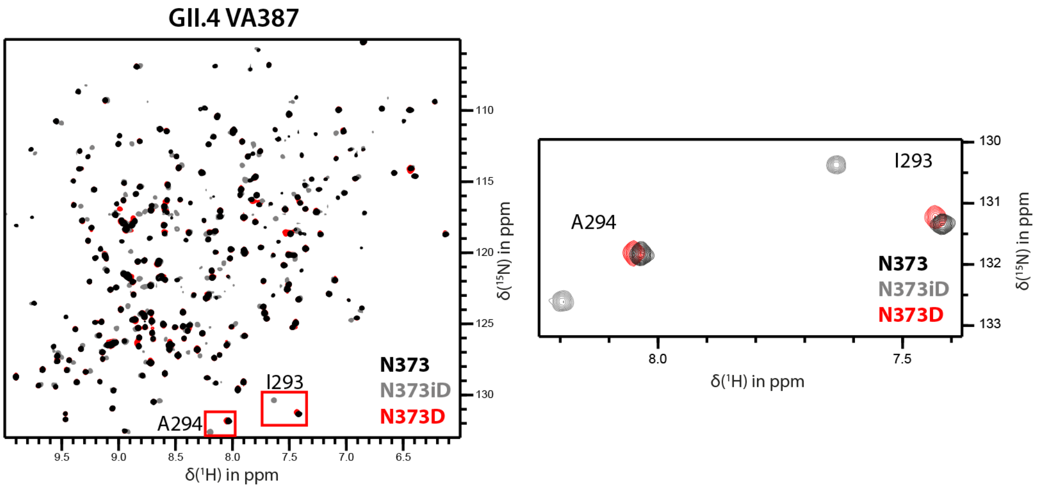


**Figure S5**. Deamidation of N373 in GII.4 VA387 NoV Saga P-domain monitored by $^{15}$N TROSY HSQC spectra. The spectrum of the fresh wild type protein is shown in black, of the point mutant N373D in red, and of deamidated wild type protein containing isoAsp in grey.

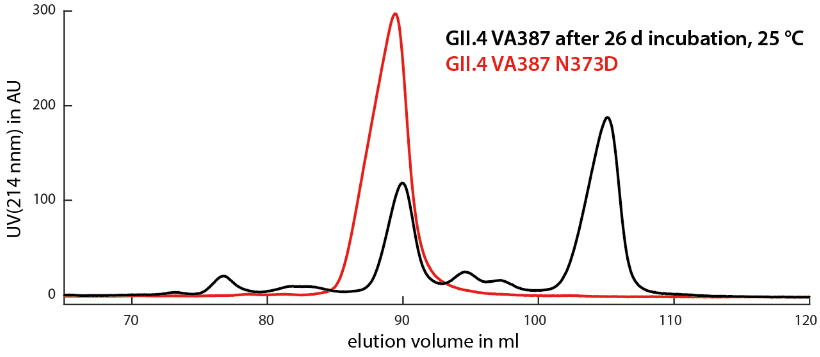


**Figure S6.** IEX behavior of the VA387 N373D mutant. Overlay of IEX chromatograms of partially deamidated wild type GII.4 VA387 (black) and the N373D mutant (red). N373D elutes at a different volume than all naturally occurring species and is, therefore, no deamidation reaction product.

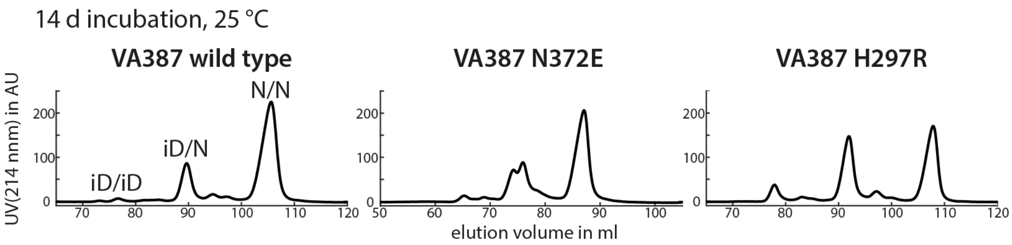


**Figure S7.** Representative IEX chromatograms of VA387 wild type and two mutant proteins N372E and H297R (cf. Fig. 3).

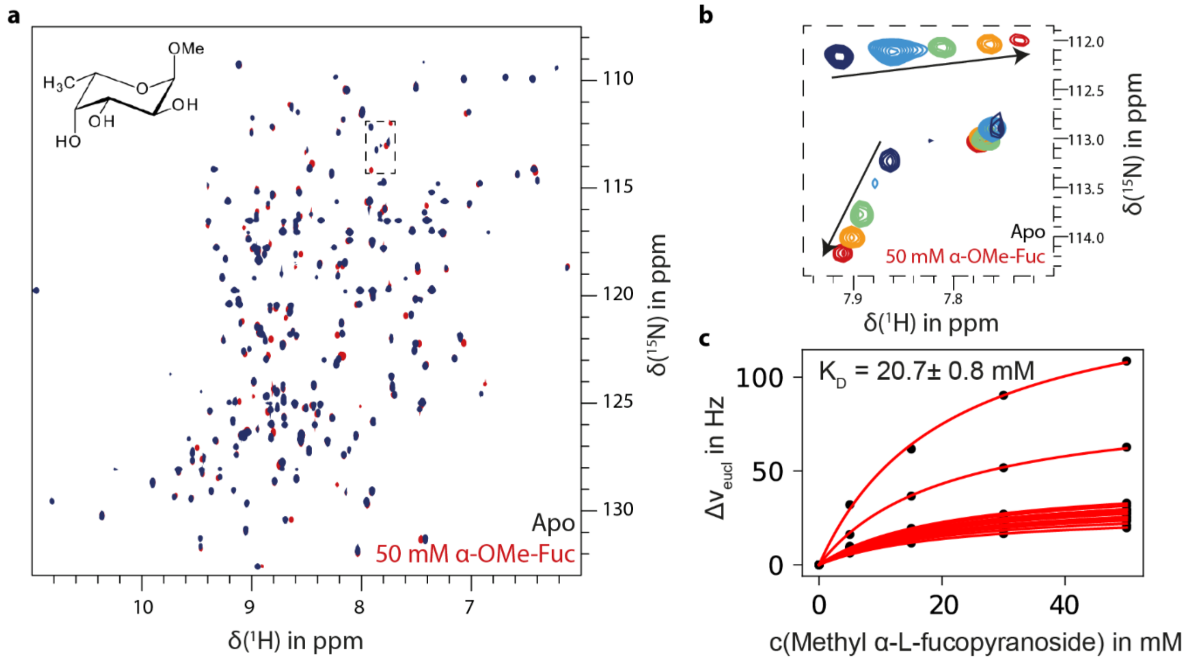


**Figure S8**. HBGA recognition of GII.4 VA387 P-domains. a, b) Titration of α-O-Me fucopyranoside to [$U$- $^{2}$H,$^{15}$N]-labeled P-domains causes Euclidean chemical shift perturbations (CSPs) that reflect binding site occupation at different total ligand concentrations. c) CSPs larger than the mean + 2 standard deviations at the highest ligand concentration were used for global fitting against the law of mass action to yield the dissociation constant $K_D$. The affinity towards fucose is identical to that found previously for GII.4 Saga P-domains[1].

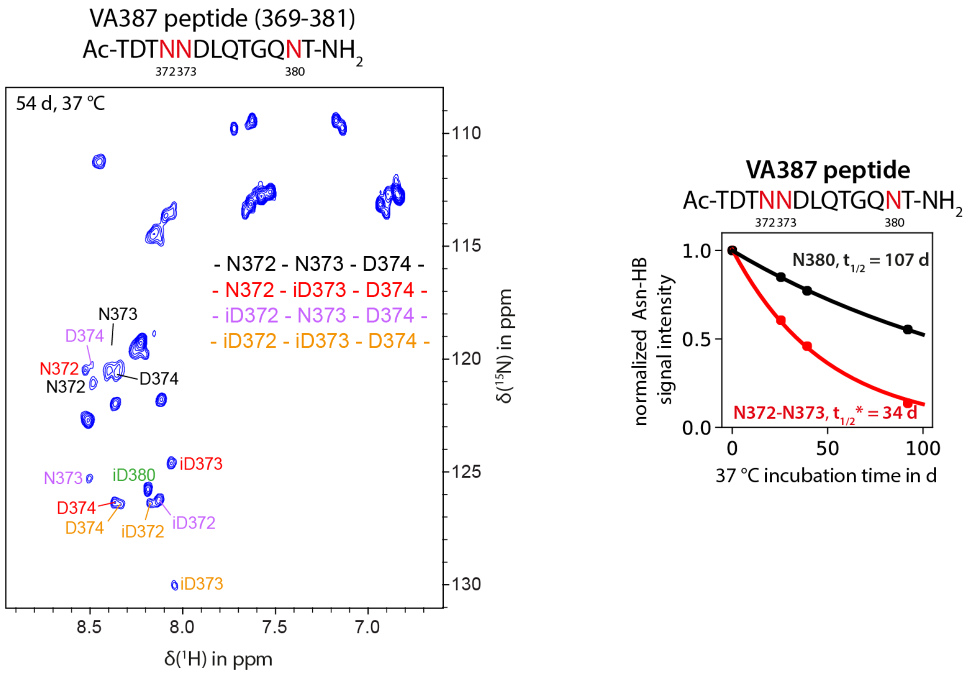


**Figure S9**. Monitoring of the deamidation of a 13-mer peptide harboring the sequence of the deamidation loop of the GII.4 VA387 NoV P-domain with 2D NMR spectroscopy. Chemical shift assignments are given in Tab. S4.

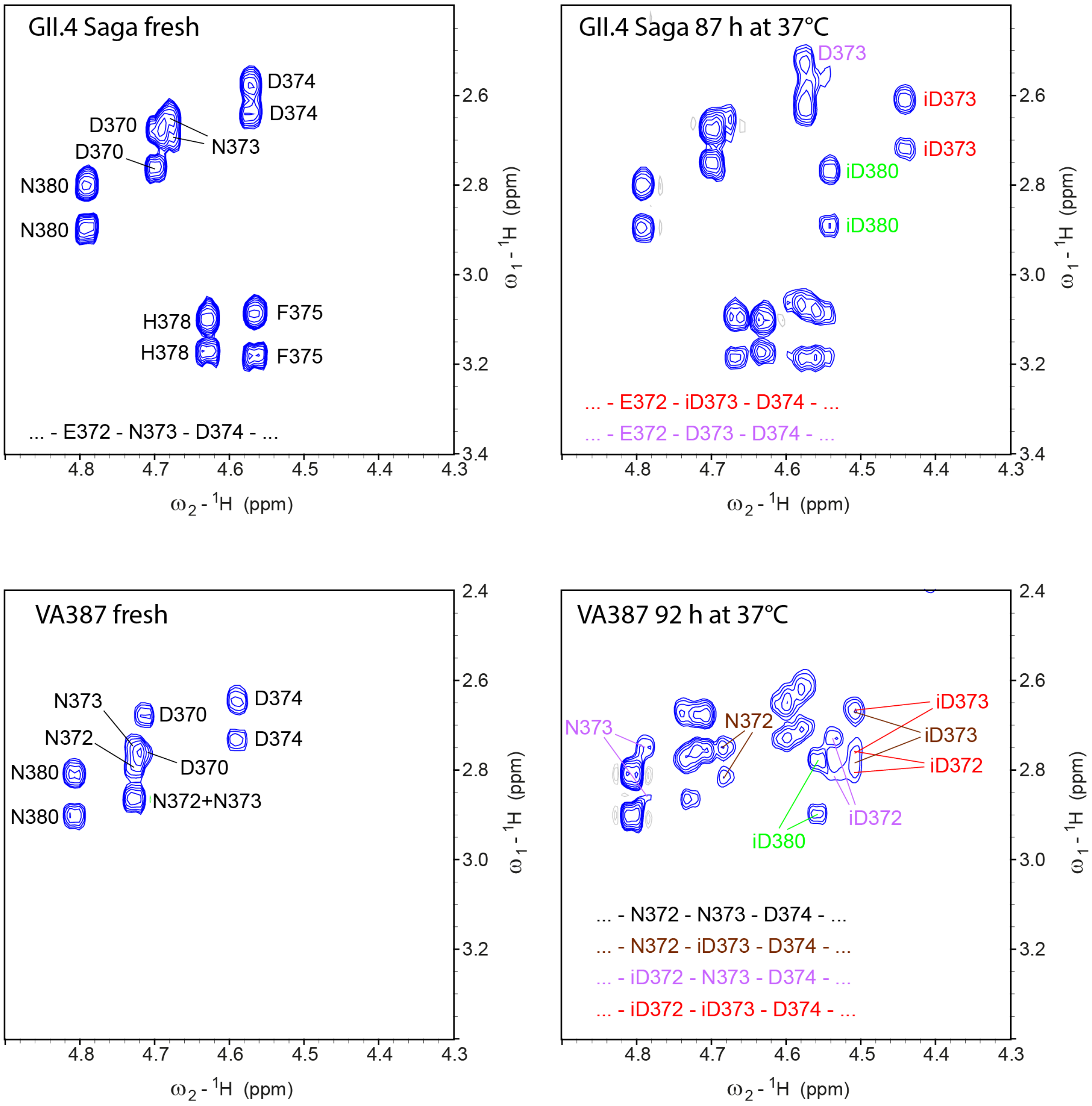


**Figure S10.** TOCSY spectra of GII.4 Saga and VA387 model peptides before and after incubation at 37 °C. Asn decay rates were quantified based on the respective HB signal intensities. Chemical shift assignments are given in Tab. S3 and S4.

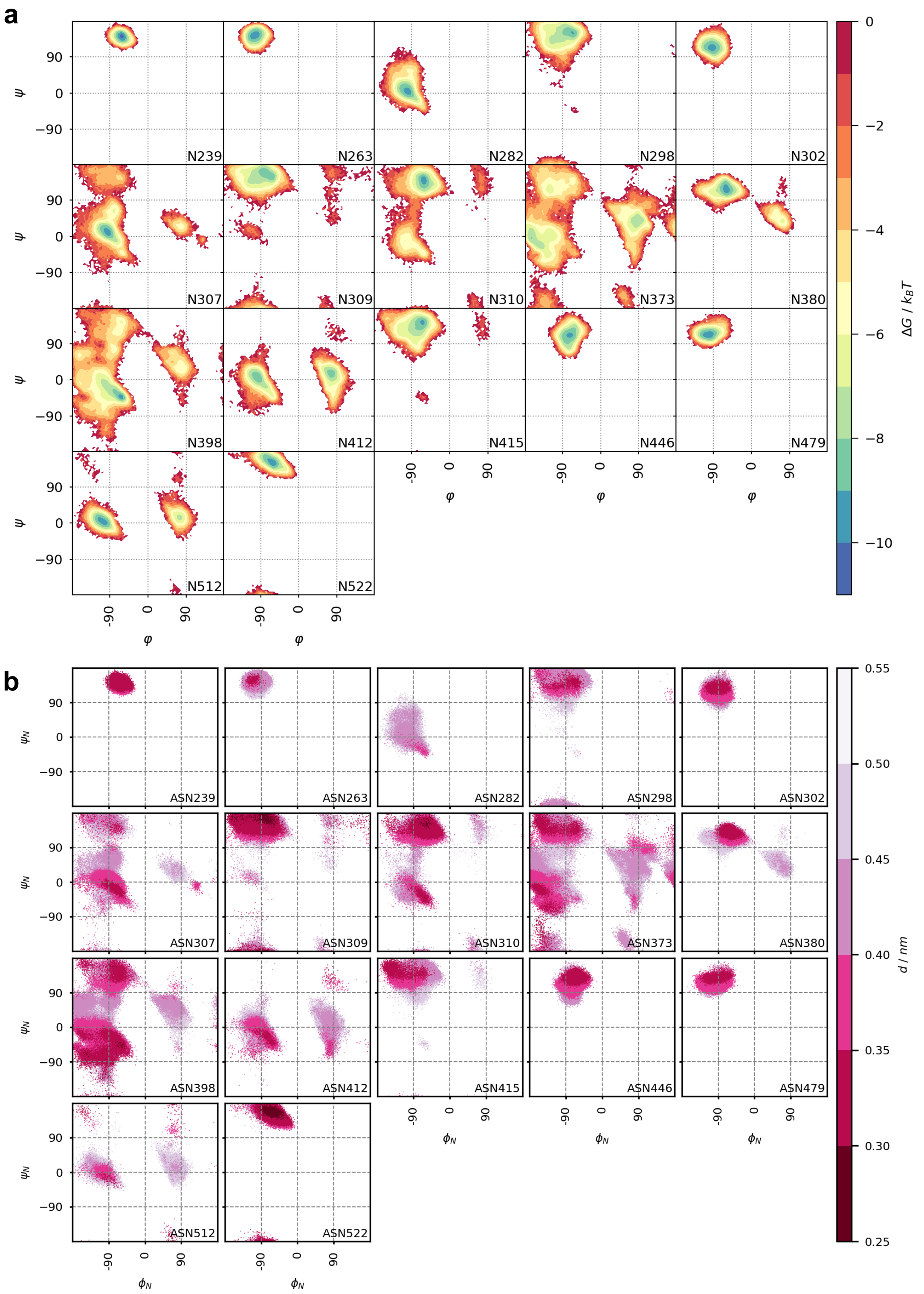


**Figure S11. a**) Free energy maps of the backbone torsion angles φ and ψ for all Asn residues in the SAGA P-dimer. **b**) Scatter plot of the backbone torsion angles, in which the color codes the nucleophilic attack distances. The color scales apply to all panels. The data was pooled from 5 individual replica simulations of 1 µs sampling time each. The backbone *syn* conformation belongs to φ, ψ values of around -180°, 0°. It is well populated an allows close distances.

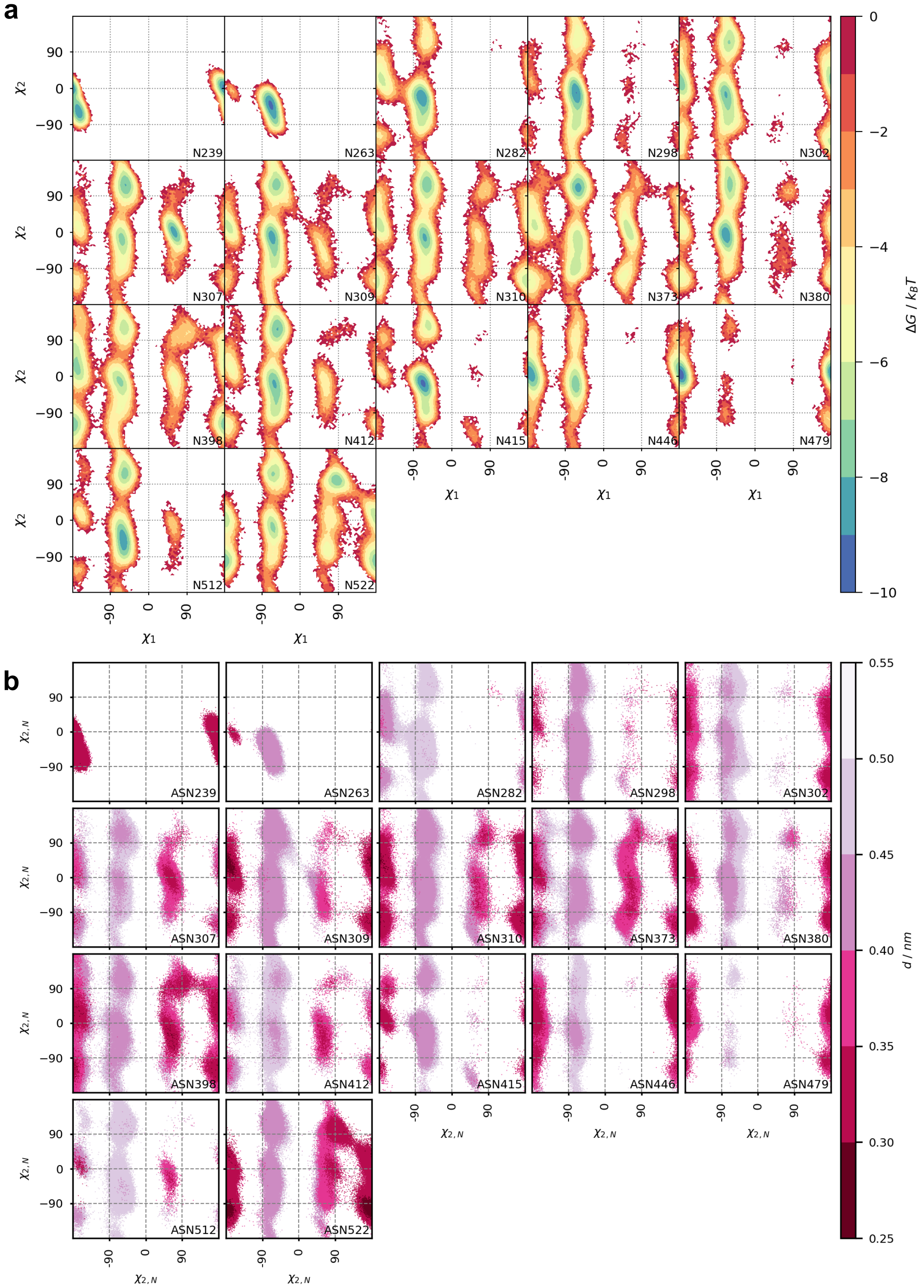


**Figure S12.** a) Free energy maps of the sidechain torsion angles $\chi_1$ and $\chi_2$ for all Asn residues in the SAGA P-dimer. b) Scatter plot of the side chains torsion angles, in which the color codes the nucleophilic attack distances. The color scales apply to all panels. The data was pooled from 5 individual replica simulations of 1 μs sampling time each. Short attack distances are frequently observed for $\chi_2$ angles of ~60° and ~180°.

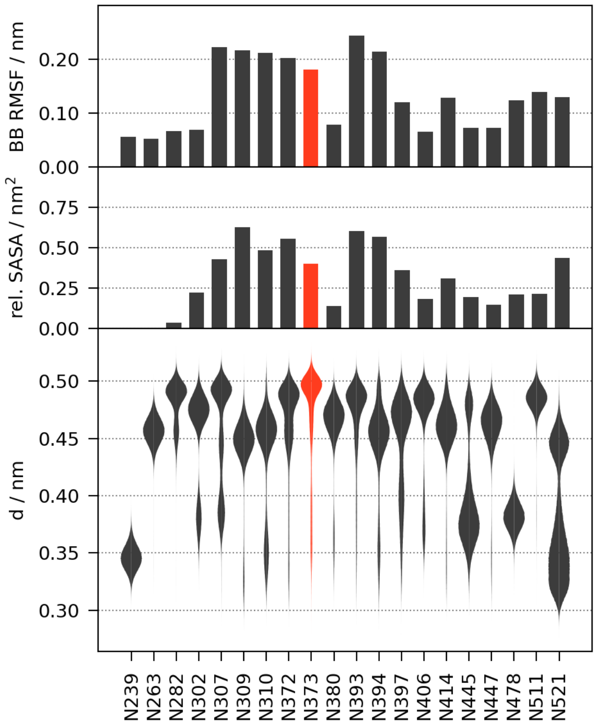


**Figure S13.** Comparison of standard deamidation descriptors for all ASNs in the VA387 P-Dimer. ASN373 Is highlighted in red. The top panel shows the average backbone RMSF. In the central panel, the average relative solvent accessibility (0: buried - 1: maximum solvent accessible) of all the ASN residues. The bottom panel shows the probability density functions of the N-CG attack distances in violin representation. The density functions are estimated using 200 bins and scaled by the maximum probability (areas are not equal to 1). The averages are computed as the means of the replica trajectory means. The density is computed from the pooled data of all trajectories.

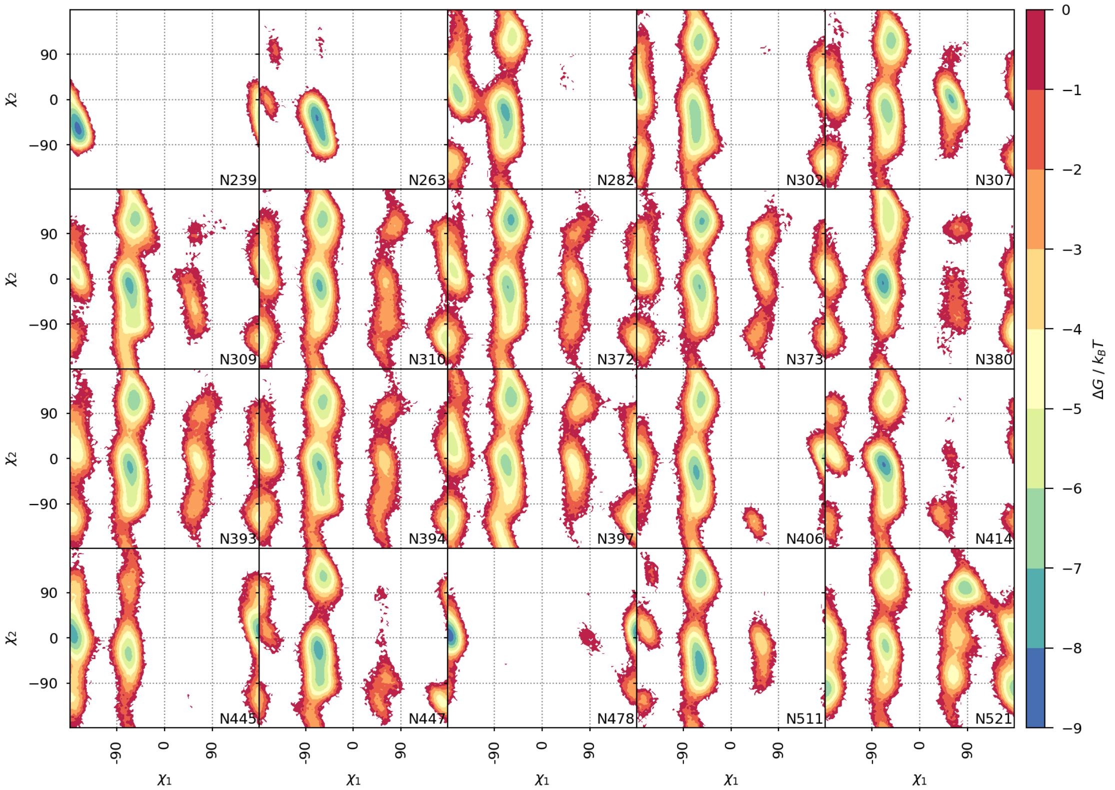


**Figure S14.** Free energy maps of the sidechain torsion angles $\chi_1$ and $\chi_2$ for all Asn residues in the VA387 P-dimer. The color scale applies to all panels. The data was pooled from 5 individual replica simulations of 1 μs sampling time each.

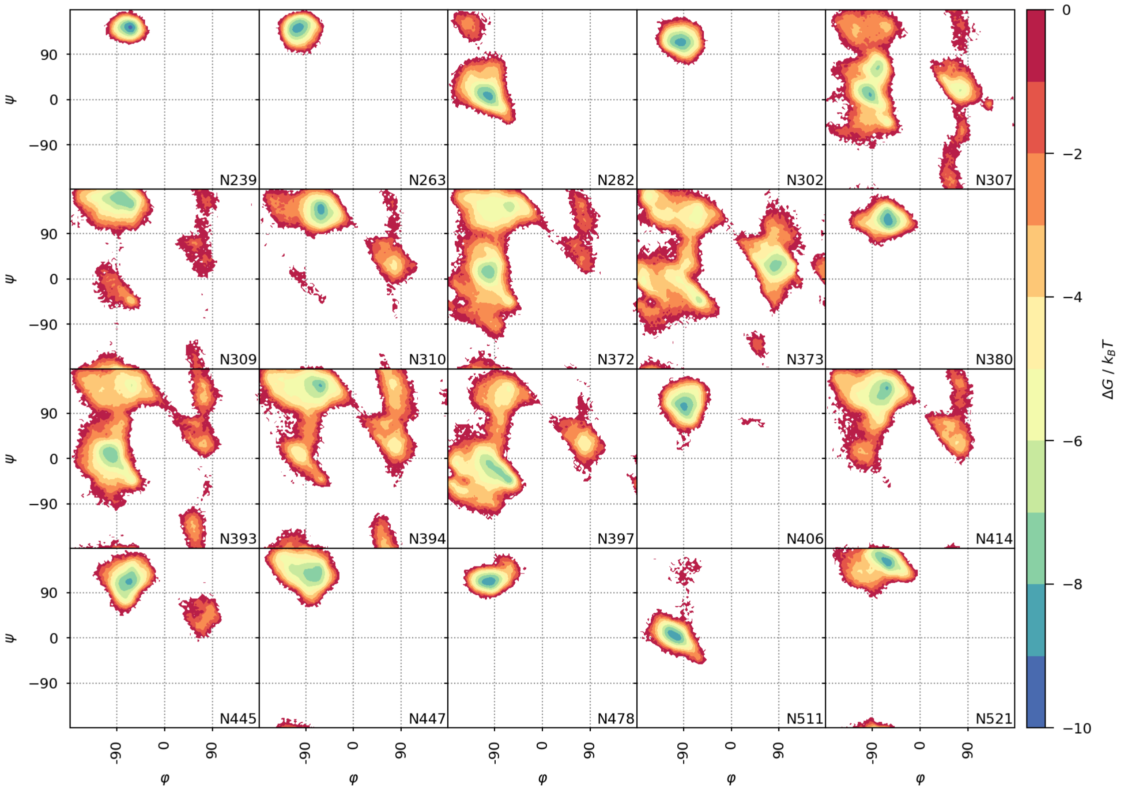


**Figure S15.** Free energy maps of the backbone torsion angles φ and ψ for all Asn residues in the VA387 P-dimer. The color scale applies to all panels. The data was pooled from 5 individual replica simulations of 1 μs sampling time each.

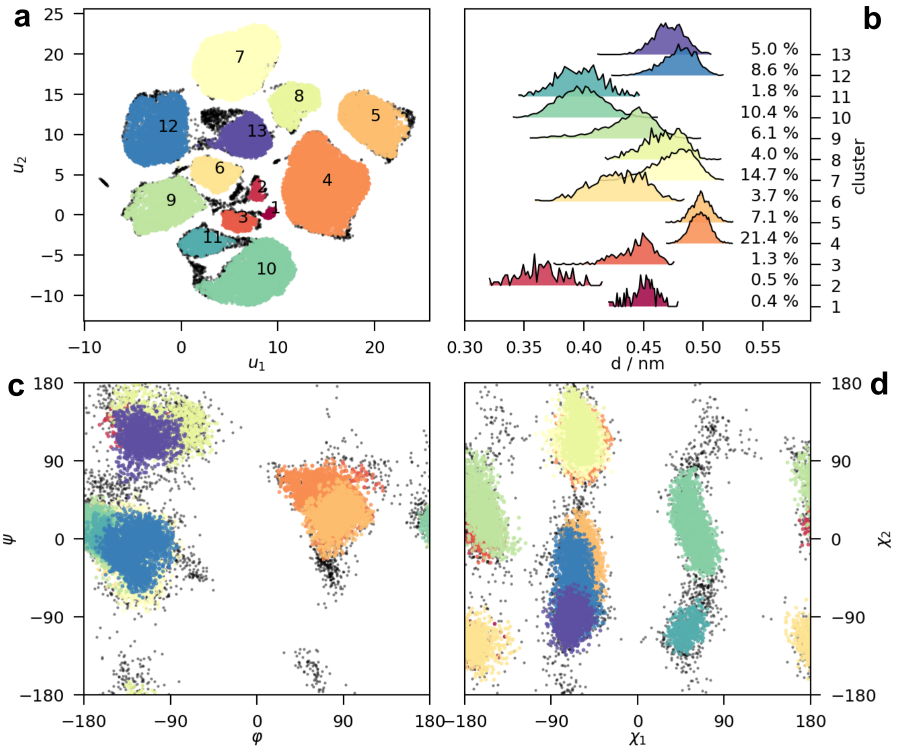


**Figure S16**. Conformational clustering of the torsion angles of Asn373 in SAGA **a)** Conformations in a UMAP 2D embedding. Noise, i.e. low energy conformations, is explicitly shown in gray and considered in further analysis. **b**) Attack distance distributions of each conformational cluster. C. Backbone torsion angles φ and ψ **d**). Sidechain torsion angles $\chi_1$ and $\chi_2$. The colors correspond to the different identified clusters and are consistent among the four panels. Clusters 9, 10, and 11 allow short attack distances and simultaneous *syn* backbone conformation.

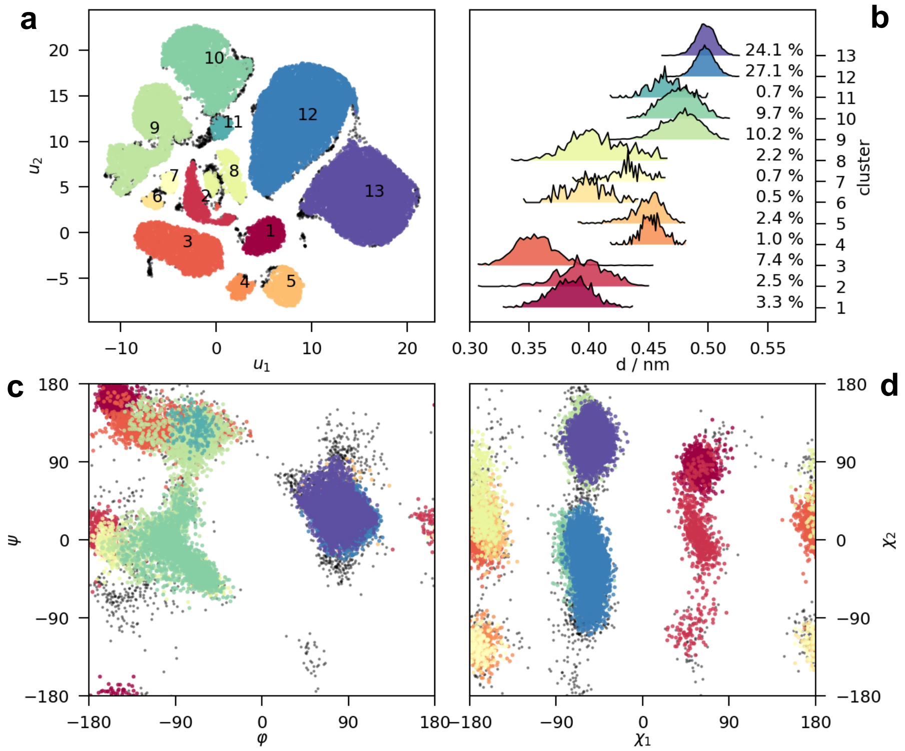


**Figure S17**. Conformational clustering of the torsion angles of Asn373 in VA387 **a)** Conformations in a UMAP 2D embedding. Noise, i.e. low energy conformations, is explicitly shown in gray and considered in further analysis. **b**) Attack distance distributions of each conformational cluster. C. Backbone torsion angles φ and ψ **d**). Sidechain torsion angles $\chi_1$ and $\chi_2$. The colors correspond to the different identified clusters and are consistent among the four panels. Clusters 2, and 8 allow short attack distances and simultaneous *syn* backbone conformation.

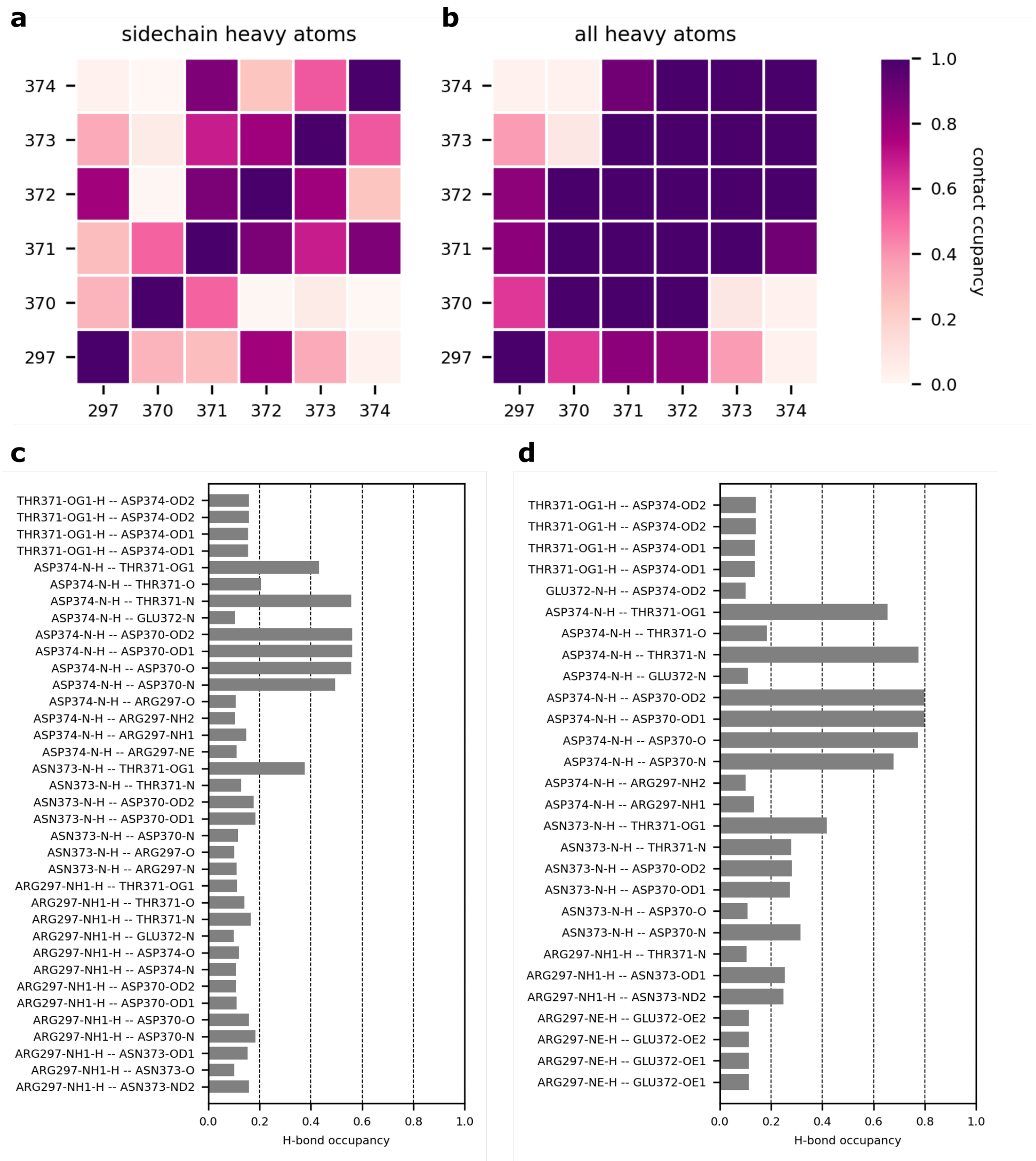


**Figure S18:** Interaction (heavy-atom distance < 0.55 nm) and hydrogen bond (donor hydrogen to acceptor distance ≤ 0.3 nm, angle ≥ 150°) occupancies between selected residues of GII.4. Saga a) Only sidechain atoms are considered. b) All atoms are considered. c) Hydrogen bonds identified in chain A of the P-dimer, averaged over the five replicates. d) Hydrogen bonds identified in chain B of the P-dimer, averaged over the five replicates. Hydrogen bonds are only shown for occupancies ≥ 0.1.

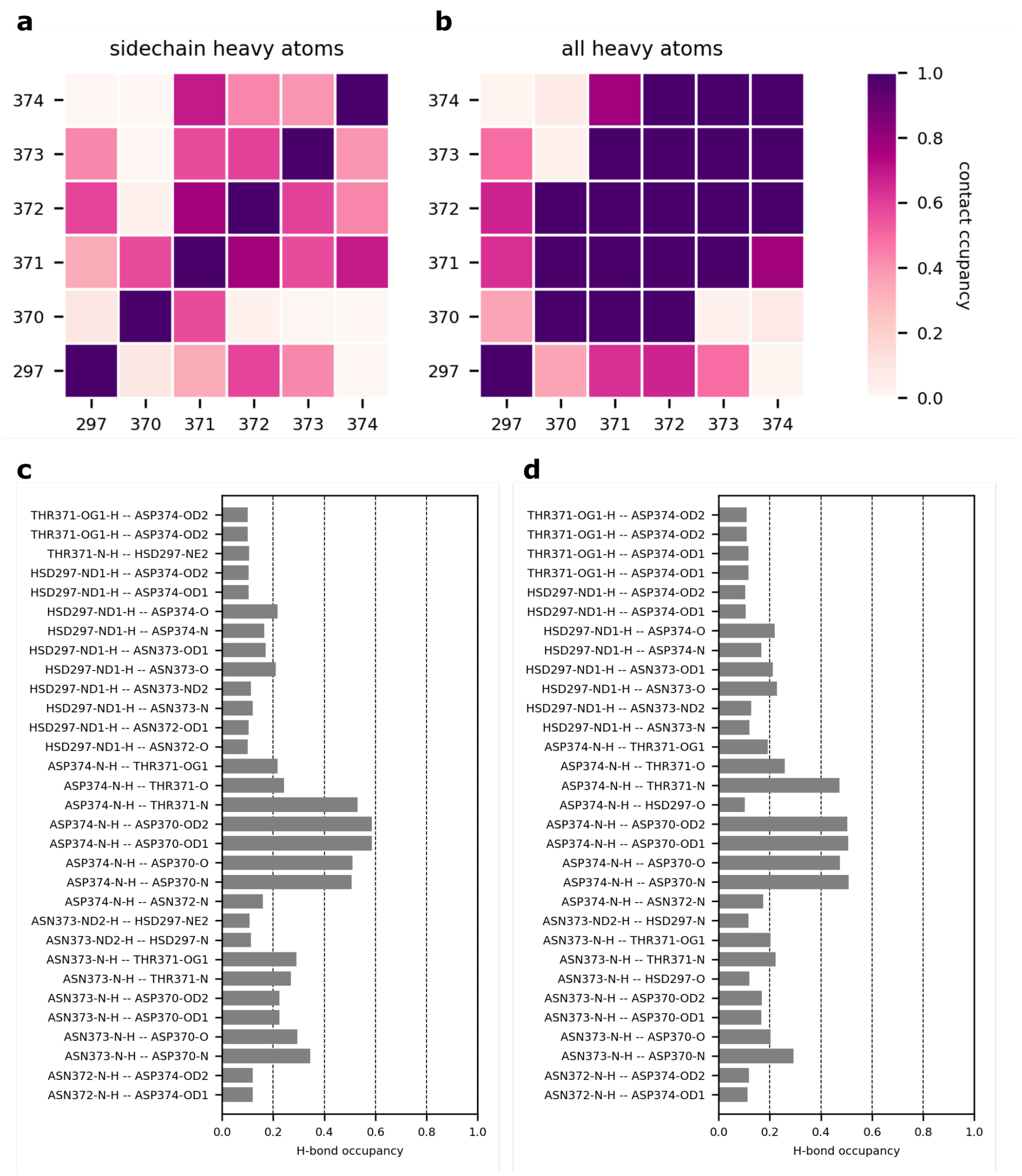


**Figure S19**: Interaction (heavy-atom distance < 0.55 nm) and hydrogen bond (donor hydrogen to acceptor distance ≤ 0.3 nm, angle ≥ 150°) occupancies between selected residues of VA387 a) Only sidechain atoms are considered. b) All atoms are considered. c) Hydrogen bonds identified in chain A of the P-dimer, averaged over the five replicates. d) Hydrogen bonds identified in chain B of the P-dimer, averaged over the five replicates. Hydrogen bonds are only shown for occupancies ≥ 0.1.

**Table S1.** Protein NMR sample compositions.

| | | | |
|---|---|---|---|
| GII.4 VA387 N/N | Fig. S3<br>Fig. S4<br>Fig. S5 | 600 MHz | 80 µM P-domain<br>500 µM DSS-$d_6$<br>in 75 mM sodium phosphate,<br>100 mM NaCl (pH 7.3)<br>10% $D_2O$ |
| GII.4 VA387 iD/iD | Fig. S4 | 600 MHz | 9 µM P-domain<br>500 µM DSS-$d_6$<br>in 75 mM sodium phosphate,<br>100 mM NaCl (pH 7.3)<br>10% $D_2O$ |
| GII.4 VA387 N373D | Fig. S5 | 600 MHz | 175 µM P-domain<br>200 µM DSS-$d_6$<br>300 µM imidazole<br>in 75 mM sodium phosphate,<br>100 mM NaCl (pH 7.3)<br>10% $D_2O$ |
| GII.4 Saga N/N | Fig. 1b | 500 MHz | 85 µM P-domain<br>100 µM DSS-$d_6$<br>300 µM imidazole<br>in 75 mM sodium phosphate,<br>100 mM NaCl (pH 7.3)<br>8% $D_2O$ |
| GII.4 Saga N/N | Fig. S3 | 600 MHz | 100 µM P-domain<br>200 µM DSS-$d_6$<br>300 µM imidazole<br>in 75 mM sodium phosphate,<br>100 mM NaCl (pH 7,3)<br>10 % D2O |
| GII.4 Saga iD/iD | Fig. 1b | 500 MHz | 105 µM P-domain<br>100 µM DSS-$d_6$<br>300 µM imidazole<br>in 75 mM sodium phosphate,<br>100 mM NaCl (pH 7.3)<br>8% $D_2O$ |
| GII.4 Saga N373D | Fig. 1b | 500 MHz | 120 µM P-domain<br>200 µM DSS-$d_6$<br>300 µM imidazole<br>in 75 mM sodium phosphate,<br>100 mM NaCl (pH 7.3)<br>10% $D_2O$ |

**Table S2.** Chemical shift assignment of GII.4 VA387 P-domains obtained by comparison of $^{15}$N HSQC spectra with those of previously assigned GII.4 Saga P-domains [1] (cf. Fig. S3). NMR samples were prepared in 75 mM sodium phosphate buffer, 100 mM NaCl, pH 7.3.

| amino acid | $^{1}$H in ppm | $^{15}$N in ppm | amino acid | $^{1}$H in ppm | $^{15}$N in ppm |
|---|---|---|---|---|---|
| 225Lys | 8.31 | 126.81 | 452Leu | 6.49 | 110.75 |
| 232Leu | 6.13 | 118.71 | 455Glu | 9.90 | 118.08 |
| 234Val | 9.20 | 120.37 | 457Val | 6.87 | 119.66 |
| 238Ser | 10.96 | 119.79 | 459His | 7.64 | 120.17 |
| 241Arg | 9.78 | 120.21 | 461Tyr | 8.25 | 117.74 |
| 242Phe | 7.39 | 121.25 | 463Glu | 8.41 | 119.12 |
| 252Gly | 6.69 | 109.87 | 464Ala | 8.34 | 122.98 |
| 259Val | 8.59 | 128.49 | 465Ala | 9.57 | 126.58 |
| 265Arg | 8.97 | 119.21 | 467Ala | 8.55 | 126.75 |
| 266Cys | 7.89 | 125.85 | 468Gln | 3.58 | 119.38 |
| 267Thr | 8.71 | 116.70 | 469Ser | 6.58 | 114.39 |
| 270Gly | 7.84 | 107.11 | 470Asp | 8.27 | 114.37 |
| 272Leu | 8.16 | 127.41 | 471Val | 8.21 | 119.07 |
| 274Gly | 9.12 | 109.28 | 472Ala | 9.05 | 130.94 |
| 282Asn | 6.84 | 117.20 | 474Leu | 8.95 | 126.22 |
| 283Ile | 6.86 | 123.90 | 475Arg | 9.02 | 122.44 |
| 285Thr | 7.09 | 103.23 | 476Phe | 8.43 | 126.02 |
| 288Gly | 8.84 | 106.91 | 477Val | 8.11 | 127.18 |
| 289Asp | 8.18 | 121.27 | 480Asp | 8.01 | 119.77 |
| 290Val | 9.77 | 112.72 | 481Thr | 6.85 | 105.19 |
| 293Ile | 7.42 | 131.35 | 483Arg | 7.51 | 120.15 |
| 294Ala | 8.04 | 131.85 | 484Val | 8.43 | 124.18 |
| 305Ser | 8.47 | 114.03 | 485Leu | 9.47 | 131.31 |
| 309Asn | 7.75 | 119.62 | 486Phe | 7.27 | 111.65 |
| 310Asn | 8.60 | 118.66 | 487Glu | 9.03 | 117.33 |
| 312Asp | 7.95 | 129.61 | 488Cys | 9.40 | 114.96 |
| 315Glu | 6.91 | 124.57 | 490Leu | 9.38 | 125.39 |
| 326Phe | 6.44 | 109.97 | 491His | 9.04 | 129.68 |
| 327Val | 7.42 | 123.28 | 497Thr | 9.35 | 114.11 |
| 330Ile | 8.72 | 124.21 | 498Val | 9.00 | 112.36 |
| 363Gly | 7.77 | 105.76 | 507Val | 8.55 | 126.49 |
| 376Gln | 8.98 | 123.98 | 512Gly | 7.68 | 106.82 |
| 387Gly | 8.11 | 104.26 | 514Phe | 8.71 | 117.01 |
| 388Val | 8.81 | 112.15 | 516Phe | 8.82 | 125.97 |
| 403Val | 7.47 | 125.25 | 517Asp | 8.14 | 129.17 |
| 404Leu | 8.05 | 126.49 | 518Ser | 6.22 | 109.34 |
| 418Ala | 10.82 | 129.58 | 519Trp | 8.71 | 124.80 |
| 428Glu | 7.54 | 116.95 | 520Val | 8.88 | 118.06 |
| 429Gln | 9.24 | 118.25 | 521Asn | 7.90 | 117.51 |
| 432Phe | 9.26 | 122.02 | 522Gln | 8.85 | 115.88 |
| 433Phe | 8.59 | 121.91 | 523Phe | 7.80 | 117.85 |
| 434Arg | 9.16 | 129.06 | 524Tyr | 7.15 | 122.90 |
| 435Ser | 8.72 | 122.32 | 525Thr | 7.44 | 124.94 |
| 449Asp | 9.46 | 131.71 | 526Leu | 9.37 | 129.11 |
| 450Cys | 9.76 | 117.51 | | | |

**Table S3** Chemical shift assignments of the GII.4 Saga peptide (369-381) in different deamidation states

| Residue | HN/N | HA/CA | HB/CB | HG/CG | HD2/ND2 |
|---|---|---|---|---|---|
| - E372-N373-D374- | | | | | |
| E372 | 8.39 / 122.7 | 4.28 / 56.8 | 2.06, 1.95 / 30.1 | 2.28, 2.24 / 36.3 | |
| N373 | 8.33 / 119.2 | 4.67 / 53.2 | 2.69, 2.65 / 39.2 | | 7.62, 6.89 / 113.5 |
| D374 | 8.25 / 121.0 | 4.57 / 54.4 | 2.63, 2.58 / 41.0 | | |
| - E372-iD373-D374- | | | | | |
| E372 | 8.37 / 123.2 | 4.35 / 56.4 | 2.10, 1.94 / n.a.[a] | 2.28, 2.23 / n.a. | |
| iD373 | 8.11 / 125.6 | 4.44 / 54.8 | 2.70, 2.60 / 40.8 | | |
| D374 | 8.21 / 126.1 | 4.55 / 54.6 | 2.60, 2.51/ n.a. | | |
| - E372-D373-D374- | | | | | |
| E372 | 8.43 /123.0 | 4.29 / n.a. | 2.06, 1.93 / n.a. | 2.25 / n.a. | |
| D373 | 8.29 / 121.4 | 4.53 / n.a. | 2.55 / n.a. | | |
| D374 | 8.36 / 121.4[b] | n.a. / n.a. | n.a. / n.a. | | |

[a] n.a. stands for not assigned
[b] both values ambigous

**Table S4.** Chemical shift assignments of the VA387 peptide (369-381) at different deamidation states

| Residue | HN/N | HA/CA | HB/CB | HG/CG | HD21, HD22/ND2 |
|---|---|---|---|---|---|
| - N372-N373-D374- | | | | | |
| N372 | 8.48 / 120.9 | 4.73 / 53.4 | 2.86, 2.79 / 38.8 | | 7.67, 6.93 / 113.3 |
| N373 | 8.40 / 119.7 | 4.72 / 53.4 | 2.85, 2.75 / 38.9 | | 7.62, 6.89 / 112.9 |
| D374 | 8.35 / 120.7 | 4.59 / 54.7 | 2.73, 2.65 / 40.9 | | |
| - N372-iD373-D374- | | | | | |
| N372 | 8.51 / 120.5 | 4.68 / 53.5 | 2.82, 2.75 / 39.1 | | n.a. / n.a.[a] |
| iD373 | 8.06 / 124.6 | 4.51 / 54.9 | 2.79, 2.67 / 40.9 | | |
| D374 | 8.37 / 126.4 | 4.58 / 54.7 | 2.70, 2.62 / 41.3 | | |
| - iD372-N373-D374- | | | | | |
| iD372 | 8.13 / 126.2 | 4.53 / 55.1 | 2.80, 2.73 / 40.8 | | |
| N373 | 8.50 / 125.3 | 4.79 / 53.3 | 2.86, 2.75 / 39.1 | | n.a. / n.a. |
| D374 | 8.50 / 120.3[b] | 4.61 / 54.7 | 2.74, 2.65 / 41.0 | | |
| - iD372-iD373-D374- | | | | | |
| iD372 | 8.18 / 126.4 | 4.51 / 55.1 | 2.82, 2.75 / 40.6 | | |
| iD373 | 8.04 / 130.0 | 4.50 / 54.8 | 2.74, 2.67 / 40.7 | | |
| D374 | 8.34 / 126.3 | 4.60 / 54.4 | 2.71, 2.60 / 41.4 | | |

[a] n.a. stands for not assigned
[b] both values ambiguous

**Table S5.** RMSF, SASA and probabilities to adopt conformations favorable for nucleophilic attack for SAGA P-dimer. The employed intervals for the angles $\alpha_{BD}$, $\alpha_{FL}$, φ and ψ are 90° ± 45°, 0° ± 45° (or 180° ± 45°), 0° ± 45° and 180° ± 60°, respectively. ∩ denotes the joint probability.

| | | | Attack geometry | | | |
|---|---|---|---|---|---|---|
| **Residue** | **RMSF/nm** | **rel. SASA** | **d<0.4nm** | **$\alpha_{BD}$≈90°** | **$\alpha_{FL}$≈0°\|180°** | **∩** |
| N239 | 0.048 | 0.001 | 1.000 | 0.985 | 0.101 | 0.101 |
| N263 | 0.048 | 0.001 | 0.001 | 1.000 | 0.000 | 0.000 |
| N282 | 0.058 | 0.084 | 0.001 | 0.993 | 0.006 | 0.000 |
| N298 | 0.102 | 0.496 | 0.007 | 0.954 | 0.003 | 0.001 |
| N302 | 0.064 | 0.187 | 0.313 | 0.873 | 0.062 | 0.050 |
| N307 | 0.183 | 0.346 | 0.314 | 0.974 | 0.036 | 0.018 |
| N309 | 0.183 | 0.481 | 0.094 | 0.822 | 0.023 | 0.014 |
| N310 | 0.188 | 0.583 | 0.221 | 0.745 | 0.120 | 0.105 |
| **N373** | **0.143** | **0.584** | **0.119** | **0.879** | **0.085** | **0.026** |
| N380 | 0.080 | 0.226 | 0.070 | 0.827 | 0.037 | 0.032 |
| N398 | 0.109 | 0.561 | 0.332 | 0.938 | 0.195 | 0.125 |
| N412 | 0.145 | 0.825 | 0.023 | 0.972 | 0.029 | 0.012 |
| N415 | 0.103 | 0.509 | 0.015 | 0.942 | 0.002 | 0.002 |
| N446 | 0.058 | 0.106 | 0.552 | 0.981 | 0.008 | 0.006 |
| N479 | 0.123 | 0.089 | 0.921 | 0.995 | 0.006 | 0.006 |
| N512 | 0.126 | 0.478 | 0.005 | 0.984 | 0.011 | 0.002 |
| N522 | 0.121 | 0.302 | 0.647 | 0.858 | 0.301 | 0.279 |

**Table S6.** RMSF, SASA and probabilities to adopt conformations favorable for nucleophilic attack for VA387 P-dimer. The employed intervals for the angles $\alpha_{BD}$, $\alpha_{FL}$, φ and ψ are 90° ± 45°, 0° ± 45° (or 180° ± 45°), 0° ± 45° and 180° ± 60°, respectively. ∩ denotes the joint probability.

| | | | **Attack geometry** | | | |
|---|---|---|---|---|---|---|
| **Residue** | **RMSF/nm** | **rel. SASA** | **d<0.4nm** | **$\alpha_{BD}$≈90°** | **$\alpha_{FL}$≈0°\|180°** | **∩** |
| N239 | 0.056 | 0.000 | 1.000 | 0.994 | 0.181 | 0.181 |
| N263 | 0.052 | 0.001 | 0.003 | 0.999 | 0.000 | 0.000 |
| N282 | 0.067 | 0.037 | 0.001 | 0.984 | 0.013 | 0.000 |
| N302 | 0.069 | 0.221 | 0.218 | 0.834 | 0.037 | 0.030 |
| N307 | 0.223 | 0.428 | 0.236 | 0.975 | 0.040 | 0.014 |
| N309 | 0.216 | 0.626 | 0.076 | 0.834 | 0.019 | 0.012 |
| N310 | 0.212 | 0.482 | 0.234 | 0.778 | 0.121 | 0.107 |
| N372 | 0.202 | 0.556 | 0.084 | 0.849 | 0.034 | 0.018 |
| **N373** | **0.181** | **0.401** | **0.140** | **0.890** | **0.034** | **0.010** |
| N380 | 0.079 | 0.138 | 0.087 | 0.868 | 0.056 | 0.051 |
| N393 | 0.244 | 0.605 | 0.084 | 0.952 | 0.044 | 0.021 |
| N394 | 0.215 | 0.566 | 0.143 | 0.824 | 0.040 | 0.028 |
| N397 | 0.121 | 0.360 | 0.193 | 0.941 | 0.131 | 0.087 |
| N406 | 0.065 | 0.184 | 0.164 | 0.852 | 0.005 | 0.002 |
| N414 | 0.129 | 0.309 | 0.047 | 0.911 | 0.009 | 0.006 |
| N445 | 0.073 | 0.193 | 0.677 | 0.988 | 0.028 | 0.026 |
| N447 | 0.073 | 0.146 | 0.041 | 0.848 | 0.028 | 0.022 |
| N478 | 0.124 | 0.211 | 0.929 | 0.998 | 0.006 | 0.006 |
| N511 | 0.139 | 0.214 | 0.014 | 0.981 | 0.015 | 0.006 |
| N521 | 0.129 | 0.435 | 0.659 | 0.876 | 0.294 | 0.272 |

**Supplementary Material and Methods Peptide Synthesis**

**Materials:** Chemical reagents and solvents for the peptide syntheses were of peptide-synthesis grade; solvents for HPLC and spectroscopy were of HPLC or spectroscopy grade. Fmoc-protected amino acids, Rink-amide MBHA resin (100-200 mesh, loading 0.57 mmol/g), N,N-diisopropylethylamine (DIPEA), piperidine, N,N-dimethylformamide (DMF), N-methyl-2-pyrrolidone (NMP), dichloromethane (DCM), diethylether and trifluoroacetic acid (TFA) were purchased from Iris Biotech (Germany). Thioanisole (TIA), acetic anhydride, acetonitrile, α-cyano-4-hydroxycinnamic acid, triisopropylsilane (TIS) and 1,2-ethanedithiol (EDT) were purchased from Sigma Aldrich (Germany). 2-(1H-benzotriazole-1-yl)-1,1,3,3-tetramethyluronium hexafluorophosphate (HBTU), N-hydroxybenzotriazole (HOBt), and N,N'-diisopropylcarbodiimide (DIC) were purchased from Biosolve (The Netherland). $D_2O$ was from Armar GmbH (Germany).

**Methods:** Solid-phase peptide synthesis was carried out on an automatic peptide synthesizer (Syro I, Biotage). The analytical HPLC equipment was from Thermo Fisher Scientific (Ultimate 3000). The analytical column was from Thermo Fisher Scientific (Syncronis $C_{18}$ , 4.6x250 mm). The gradient used for analytical HPLC was the following: 3% B for 8 min, up to 60% B over 35 min (A = $H_2O$ with 0.06% TFA; B = $CH_3CN$ with 0.05% TFA). MALDI-TOF mass spectra were recorded on an Autoflex mass spectrometer from Bruker Daltonics using α-cyano-4-hydroxycinnamic acid as matrix.

**Table S7.** Analytical characterization of the synthetic peptides used in this work.

| **Number** | **Peptide sequence** | **Mtheor.[a] (Da)** | **Mfound [b] (Da)** | **$t_R$[c] (min)** |
|---|---|---|---|---|
| **1** | *Ac*-TDTENDFETHQNT-*NH₂* | 1592.53 | 1591.84 | 19.463 |
| **2** | *Ac*-TDTNNDLQTGQNT-*NH₂* | 1462.43 | 1461.45 | 16.897 |

a. Averaged mass
b. Measured by MALDI-TOF-MS
c. HPLC gradient: 3% B for 8 min. 3-60% in 35 min. with A = 0.06% TFA in water and B = 0.05% TFA in acetonitrile

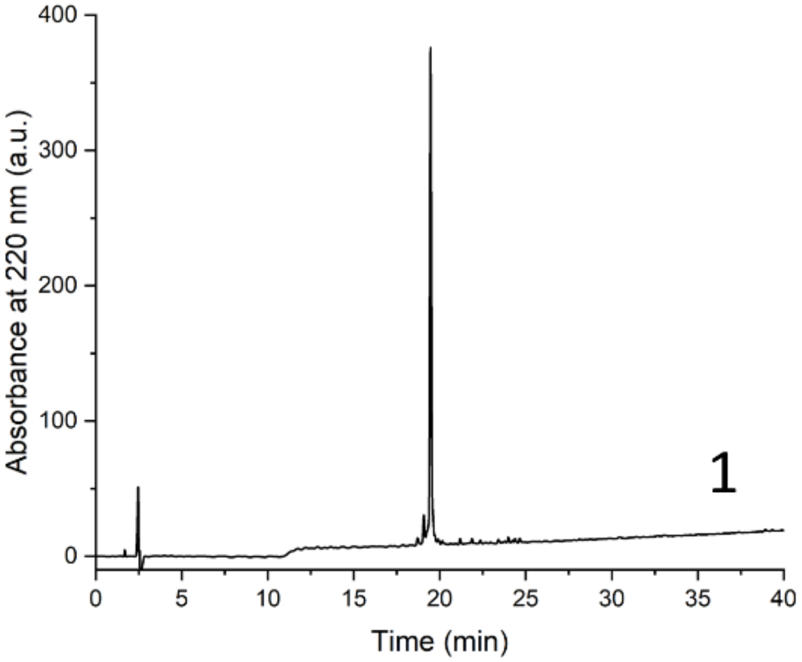


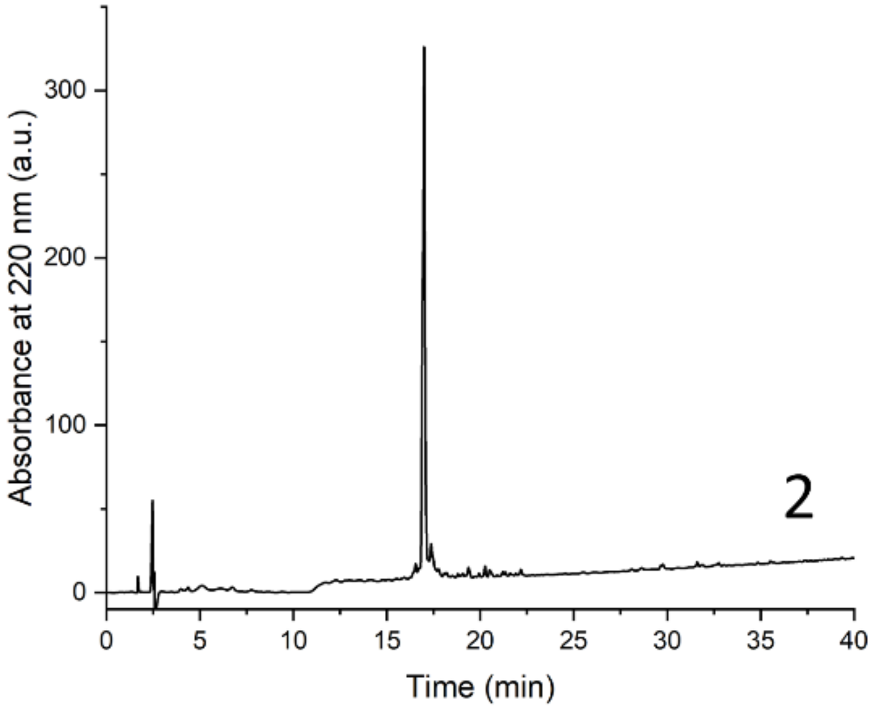


**Figure S20.** Analytical HPLC of the synthetic peptides used in this work (see Table S7 for $t_R$ values and gradient).

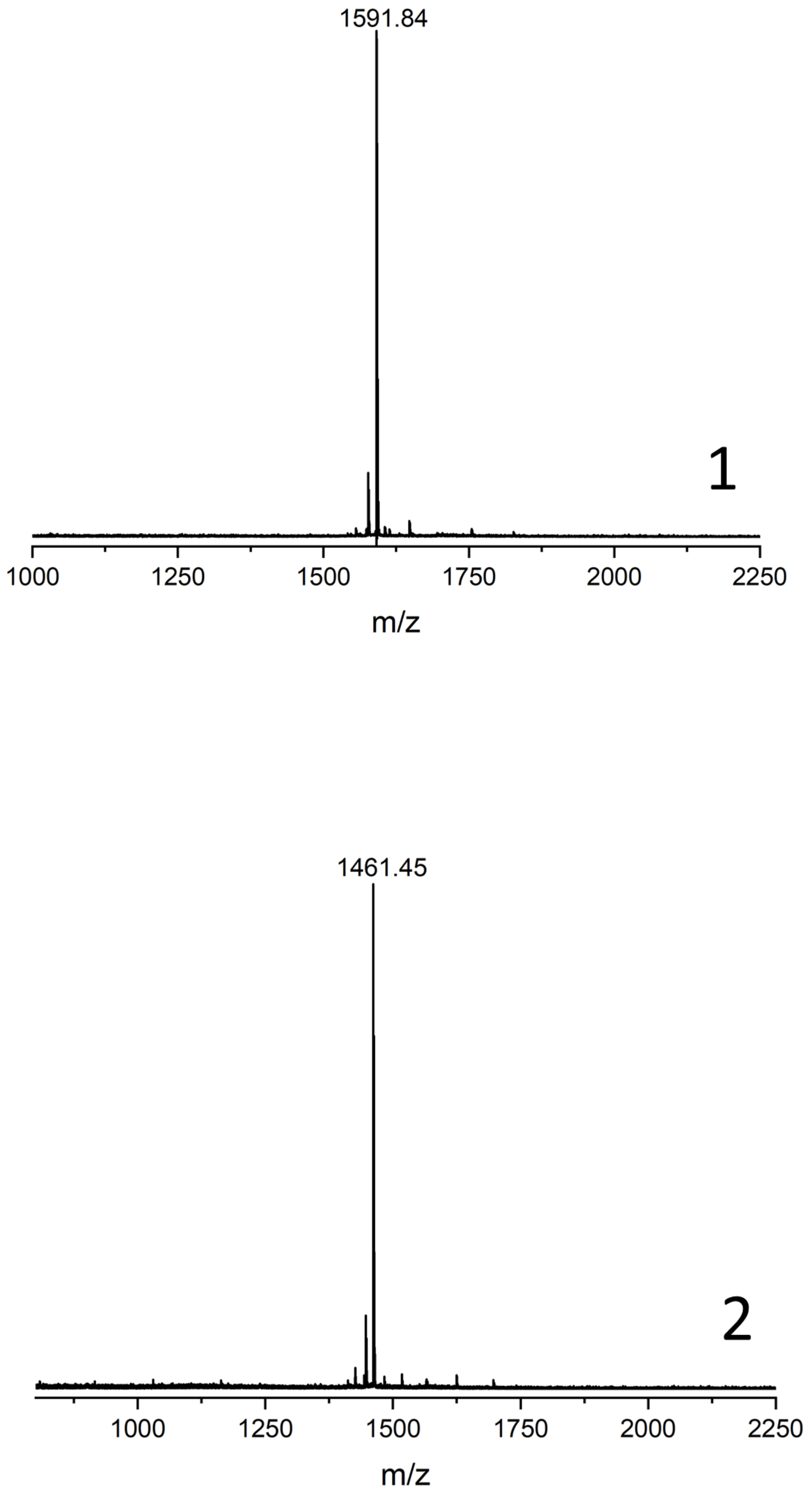


**Figure S21.** MALDI-TOF-MS of the synthetic peptides used in this work (see Table S7 for Mtheor.).

**Supplementary Methods Molecular Dynamics**

Theoretical conformational sampling was achieved using explicit-solvent, full-atomistic equilibrium molecular dynamics. Two molecular systems were subjected to molecular dynamics integration: 1. the P-protein dimer from strain GII.4 SAGA, and 2. the P-protein dimer from strain VA387. For both systems, data were collected from five trajectory replica of 1 µs length each, which were individually equilibrated using different initial velocity distributions. One Saga MD 1 µs trajectory was used previously to sample protein conformations for ensemble docking to the bile acid binding grove [2]. The SAGA P-dimer simulations also contained 5 molecules of bile acid salt, which did not form any stable interactions with the P-dimers. In both P-dimers, histidine moieties were protonated according to possible hydrogen bond formations with neighboring amino acids. In particular, histidine residues 292, 347, 417, 460, and 501 were protonated at the Nϵ position, histidines 378, 396, 414, and 490 at the Nδ. For histidine 505 we chose to protonate at both Nϵ and Nδ.

For the SAGA P-dimer, the molecular simulation tasks were performed with GROMACS 5.1.5 [3-8] using CHARMM36 force field parameters [9]. Modeling of the initial system was attained with CHARMM-GUI solvation builder [10] using the X-ray structure PDB 4OOX [11]. CHARMM-GUI was also used for solvation with TIP3P water and ionization to 0.15 M NaCl. The periodic simulation box was set to a cubic shape of 9.3x9.3x9.3 nm³ volume, corresponding to 2 nm water layers in each direction around the central protein dimer. Prior to dynamics integration, the system was minimized for 5000 steps using a steepest descent algorithm. Dynamics were initiated by assigning velocities according to a Maxwell-Boltzmann distribution at 303.15 K, followed by NVT equilibration for 200 ps (time step 0.002 ps) using Nose-Hoover [12-14] temperature coupling (coupling constant 0.4 ps-1, reference temperature 303.15 k). Protein and solvent were coupled to individual baths. To relax the box volume, 200 ps of NPT (time step 0.002 ps) sampling using an isotropic Berendsen coupling [15-16] with a reference pressure of 1 ATM and a compressibility of 4.5e-5 $ATM^{-1}$ were attached. The box volume was adjusted every 0.5 ps. During minimization and equilibration, the backbone atoms were restrained by 400 kJ/mol/nm and the sidechain atoms by 40 kJ/mol/nm. For the 1 µs of unrestrained production (time step 0.002 ps), Parinello-Rahman pressure coupling [17-18] was applied instead and the temperature coupling constant was increased to 2 ps. Snapshots were stored every 20 ps. During all the steps, covalent bonds to hydrogen atoms were constraint using LINCS [19-20] as a solver. Coulombic interactions were computed using the PME method [21-23] and a cutoff of 1.2 nm. Van-der-Waals interactions had a cutoff 1.2 nm with a force-switch modifier starting at 1.0 nm. Center of mass movement of the whole system was removed every 100 steps.

The simulation protocol was marginally updated for the VA387 P-dimer MD calculations. In particular, we here used GROMACS 2018.3 and discarded the NPT equilibration step because the long simulation time renders the initial few nanoseconds of box size equilibration negligible. However, we used an initial NVT equilibration of 125.000 steps (0.001 ps time step). Additionally, we applied restraints to the protein dihedrals during the equilibration (4.0 kJ/mol/deg). Also, the solvation box size was slightly smaller (9.2x9.2x9.2 nm³). Other parameters were identical. We note that the SAGA calculations were performed on multiple CPU nodes, whereas the VA387 simulations were achieved using single CUDA GPU nodes. However, we do not expect the adjustments to affect the overall outcome of our calculations. Regarding the length of the trajectories and the substantial computational effort, we justify the adjustments by a better utilization of compute resources.

Data analysis and visualization were carried out with GROMACS tools and the Python libraries NumPy [24-25], MDTraj [26]and MatPlotLib [27]. The root mean squared fluctuation (RMSF) was computed using gmx rmsf. Only the backbone atoms C, N, CA and O were considered. Rotational und translational motions were removed using least-square super positioning of the backbone atoms. The per-residue solvent accessible surface area (SASA) was computed with gmx sasa and probe radius of 0.14 nm and 24 dots. To calculate the relative surface accessibility of the Asn residues, we divided their absolute SASA by 1.95 nm², corresponding to the theoretical maximum SASA for Asn [28]. The sidechain torsion angles of the Asn residues, as well as the distances from the Cγ atoms of Asn to the backbone nitrogen atoms of

the subsequent amino acids were computed with MDTraj. The Asn torsion angles are defined as: φ: Ci-1-Ni-Cαi-Ci, ψ: Ni-Cαi-Ci-Ni+1, χ1: N-Cα-Cβ-Cγ, and χ2: Cα-Cβ-Cγ-Oδ (Figure 22 a). The free energy maps were constructed from the 2D probability densities as estimated by binning the data to 100 x 100 bins of $2\pi/100$ widths. The relative free energies in units of kBT are computed as the negative natural logarithm of the probability density. Clustering was performed in the cosine-sine feature space spanned by the transformation of the four torsion angles z(φ)=[cos (φ), sin(φ)]. Hierarchical density-based clustering was calculated using the HDBSAN [29] algorithm with cluster selection alpha value of 0.5, a minimum sample size of 100 and a minimum cluster size of 100 (other parameters were left default).

The Bürgi-Dunitz (BD) [30] and Flipping-Lodge (FL) [31] angles were calculated to better describe the nucleophilic attack geometry (Figure S22b). They are based on a coordinate transformation that centers the carbonyl carbon to the origin and the carbonyl plane onto the XY-plane. Then, the BD angle is defined as the angle between the vectors connecting the carbonyl carbon with carbonyl oxygen and the carbonyl carbon with nucleophile. It can be described as the as altitude angle of the nucleophile when the electrophile (carbonyl carbon) is the reference. It is between 0 and 180°. The FL angle can be imaged as the inclination angle of the nucleophile relative to the normal of the carbonyl plane. Here, it is calculated as the pseudo-torsion angle between the carbonyl plane normal vector, the carbonyl carbon to carbonyl vector and the carbonyl carbon to nucleophile angle. It defined in a way that it is positive for a rotation towards the Cβ and negative towards Nδ2. Its range is between -180° and 180°.

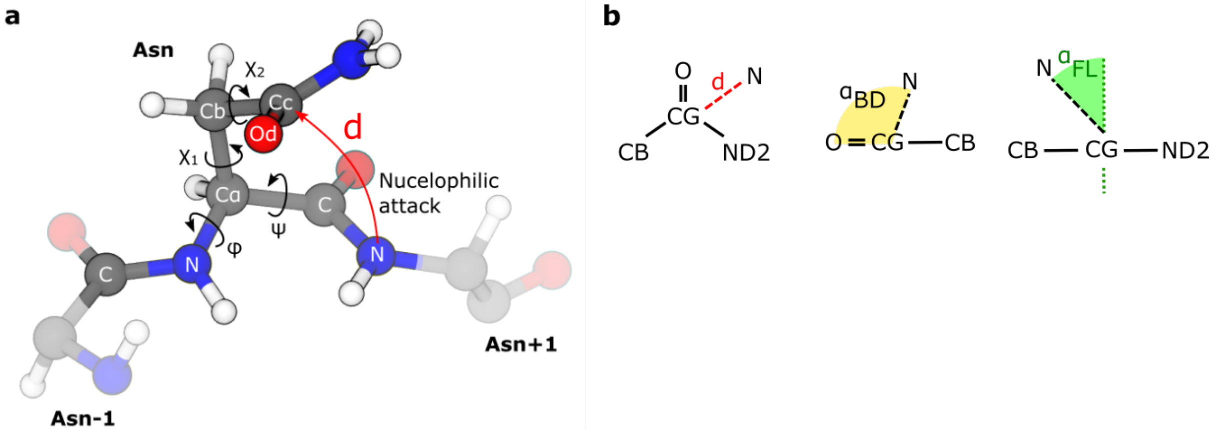


**Figure S22** Attack geometry and key conformational descriptors. a) φ: $C_{Asn-1}$-$N_{Asn}$-$C\alpha_{Asn}$-$C_{Asn}$, ψ: $N_{Asn}$-$C\alpha_{Asn}$-$C_{Asn}$-$N_{Asn+1}$, χ1: $N_{Asn}$-$C\alpha_{Asn}$-$C\beta_{Asn}$-$C\gamma_{Asn}$, χ2: $C\alpha_{Asn}$-$C\beta_{Asn}$-$C\gamma_{Asn}$-$O\delta_{Asn}$ b) Definition of d and BD and FL angles in the CG-centered coordinate system.

REFERENCES


1. Mallagaray, A.; Creutznacher, R.; Dulfer, J.; Mayer, P. H. O.; Grimm, L. L.; Orduna, J. M.; Trabjerg, E.; Stehle, T.; Rand, K. D.; Blaum, B. S.; Uetrecht, C.; Peters, T., A post-translational modification of human Norovirus capsid protein attenuates glycan binding. *Nat Commun* **2019,** *10* (1), 1320.
2. Creutznacher, R.; Schulze, E.; Wallmann, G.; Peters, T.; Stein, M.; Mallagaray, A., Chemical-Shift Perturbations Reflect Bile Acid Binding to Norovirus Coat Protein: Recognition Comes in Different Flavors. *ChemBioChem* **2020,** *21* (7), 1007-1021.
3. Berendsen, H. J. C.; van der Spoel, D.; van Drunen, R., GROMACS: A message-passing parallel molecular dynamics implementation. *Computer Physics Communications* **1995,** *91* (1), 43-56.
4. Lindahl, E.; Hess, B.; van der Spoel, D., GROMACS 3.0: a package for molecular simulation and trajectory analysis. *Molecular modeling annual* **2001,** *7* (8), 306-317.
5. Hess, B.; Kutzner, C.; van der Spoel, D.; Lindahl, E., GROMACS 4: Algorithms for Highly Efficient, Load-Balanced, and Scalable Molecular Simulation. *Journal of Chemical Theory and Computation* **2008,** *4* (3), 435-447.
6. Pronk, S.; Páll, S.; Schulz, R.; Larsson, P.; Bjelkmar, P.; Apostolov, R.; Shirts, M. R.; Smith, J. C.; Kasson, P. M.; van der Spoel, D.; Hess, B.; Lindahl, E., GROMACS 4.5: a high-throughput and highly parallel open source molecular simulation toolkit. *Bioinformatics* **2013,** *29* (7), 845-854.
7. Abraham, M. J.; Murtola, T.; Schulz, R.; Páll, S.; Smith, J. C.; Hess, B.; Lindahl, E., GROMACS: High performance molecular simulations through multi-level parallelism from laptops to supercomputers. *SoftwareX* **2015,** *1-2*, 19-25.
8. Páll, S.; Abraham, M. J.; Kutzner, C.; Hess, B.; Lindahl, E. In *Tackling Exascale Software Challenges in Molecular Dynamics Simulations with GROMACS*, Solving Software Challenges for Exascale, Cham, 2015//; Markidis, S.; Laure, E., Eds. Springer International Publishing: Cham, 2015; pp 3-27.
9. Huang, J.; MacKerell, A. D., Jr., CHARMM36 all-atom additive protein force field: validation based on comparison to NMR data. *J Comput Chem* **2013,** *34* (25), 2135-45.
10. Jo, S.; Kim, T.; Iyer, V. G.; Im, W., CHARMM-GUI: A web-based graphical user interface for CHARMM. *Journal of Computational Chemistry* **2008,** *29* (11), 1859-1865.
11. Singh, B. K.; Leuthold, M. M.; Hansman, G. S.; Sandri-Goldin, R. M., Human Noroviruses' Fondness for Histo-Blood Group Antigens. *Journal of Virology* **2015,** *89* (4), 2024-2040.
12. Rühle, V., Berendsen and nose-hoover thermostats. *Am. J. Phys* **2007**.
13. Braga, C.; Travis, K. P., A configurational temperature Nosé-Hoover thermostat. *The Journal of Chemical Physics* **2005,** *123* (13), 134101.
14. Hoover, W. G.; Holian, B. L., Kinetic moments method for the canonical ensemble distribution. *Physics Letters A* **1996,** *211* (5), 253-257.
15. Berendsen, H. J. C.; Postma, J. P. M.; van Gunsteren, W. F.; DiNola, A.; Haak, J. R., Molecular dynamics with coupling to an external bath. *The Journal of Chemical Physics* **1984,** *81* (8), 3684-3690.
16. Lemak, A.; Balabaev, N., On the Berendsen thermostat. *Molecular Simulation* **1994,** *13* (3), 177-187.
17. Parrinello, M.; Rahman, A., Crystal Structure and Pair Potentials: A Molecular-Dynamics Study. *Physical Review Letters* **1980,** *45* (14), 1196-1199.
18. Martonak, R.; Laio, A.; Parrinello, M., Predicting crystal structures: The Parrinello-Rahman method revisited. *Physical Review Letters* **2003,** *90* (7).
19. Hess, B.; Bekker, H.; Berendsen, H. J. C.; Fraaije, J. G. E. M., LINCS: A linear constraint solver for molecular simulations. *Journal of Computational Chemistry* **1997,** *18* (12), 1463-1472.
20. Hess, B., P-LINCS: A Parallel Linear Constraint Solver for Molecular Simulation. *Journal of Chemical Theory and Computation* **2008,** *4* (1), 116-122.

21. Wells, B. A.; Chaffee, A. L., Ewald Summation for Molecular Simulations. *Journal of Chemical Theory and Computation* **2015,** *11* (8), 3684-3695.
22. Yeh, I.-C.; Berkowitz, M. L., Ewald summation for systems with slab geometry. *The Journal of Chemical Physics* **1999,** *111* (7), 3155-3162.
23. Essmann, U.; Perera, L.; Berkowitz, M. L.; Darden, T.; Lee, H.; Pedersen, L. G., A smooth particle mesh Ewald method. *The Journal of Chemical Physics* **1995,** *103* (19), 8577-8593.
24. Harris, C. R.; Millman, K. J.; van der Walt, S. J.; Gommers, R.; Virtanen, P.; Cournapeau, D.; Wieser, E.; Taylor, J.; Berg, S.; Smith, N. J., Array programming with NumPy. *Nature* **2020,** *585* (7825), 357-362.
25. Van Der Walt, S.; Colbert, S. C.; Varoquaux, G., The NumPy array: a structure for efficient numerical computation. *Computing in science & engineering* **2011,** *13* (2), 22-30.
26. McGibbon, Robert T.; Beauchamp, Kyle A.; Harrigan, Matthew P.; Klein, C.; Swails, Jason M.; Hernández, Carlos X.; Schwantes, Christian R.; Wang, L.-P.; Lane, Thomas J.; Pande, Vijay S., MDTraj: A Modern Open Library for the Analysis of Molecular Dynamics Trajectories. *Biophysical Journal* **2015,** *109* (8), 1528-1532.
27. Hunter, J. D., Matplotlib: A 2D graphics environment. *Computing in science & engineering* **2007,** *9* (03), 90-95.
28. Tien, M. Z.; Meyer, A. G.; Sydykova, D. K.; Spielman, S. J.; Wilke, C. O., Maximum Allowed Solvent Accessibilites of Residues in Proteins. *PLOS ONE* **2013,** *8* (11), e80635.
29. McInnes, L.; Healy, J.; Astels, S., hdbscan: Hierarchical density based clustering. *Journal of Open Source Software* **2017,** *2* (11), 205.
30. Bürgi, H. B.; Dunitz, J. D.; Lehn, J. M.; Wipff, G., Stereochemistry of reaction paths at carbonyl centres. *Tetrahedron* **1974,** *30* (12), 1563-1572.
31. Heathcock, C. H.; Flippin, L. A., Acyclic stereoselection. 16. High diastereofacial selectivity in Lewis acid mediated additions of enol silanes to chiral aldehydes. *Journal of the American Chemical Society* **1983,** *105* (6), 1667-1668.
